# Parallel Genomics Uncover Novel Enterococcal-Bacteriophage Interactions

**DOI:** 10.1128/mBio.03120-19

**Published:** 2020-03-03

**Authors:** Anushila Chatterjee, Julia L. E. Willett, Uyen Thy Nguyen, Brendan Monogue, Kelli L. Palmer, Gary M. Dunny, Breck A. Duerkop

**Affiliations:** a Department of Immunology and Microbiology, University of Colorado School of Medicine, Aurora, Colorado, USA; b Department of Microbiology and Immunology, University of Minnesota Medical School, Minneapolis, Minnesota, USA; c Department of Biological Sciences, University of Texas at Dallas, Richardson, Texas, USA; University of Pittsburgh

**Keywords:** bacteriophages, *Enterococcus*, antibiotic resistance, transposons, RNA-Seq, Tn-Seq, phage-bacterium interactions

## Abstract

Bacteriophages (phages) have been proposed as alternative therapeutics for the treatment of multidrug-resistant bacterial infections. However, there are major gaps in our understanding of the molecular events in bacterial cells that control how bacteria respond to phage predation. Using the model organism Enterococcus faecalis, we used two distinct genomic approaches, namely, transposon library screening and RNA sequencing, to investigate the interaction of E. faecalis with a virulent phage. We discovered that a transcription factor encoding a LytR family response regulator controls the expression of enterococcal polysaccharide antigen (*epa*) genes that are involved in phage infection and bacterial fitness. In addition, we discovered that DNA mismatch repair mutants rapidly evolve phage adsorption deficiencies, underpinning a molecular basis for *epa* mutation during phage infection. Transcriptomic profiling of phage-infected E. faecalis revealed broad transcriptional changes influencing viral replication and progeny burst size. We also demonstrate that phage infection alters the expression of bacterial genes associated with intra- and interbacterial interactions, including genes involved in quorum sensing and polymicrobial competition. Together, our results suggest that phage predation has the potential to influence complex microbial behavior and may dictate how bacteria respond to external environmental stimuli. These responses could have collateral effects (positive or negative) on microbial communities, such as the host microbiota, during phage therapy.

## Abstract (precis)

We lack fundamental understanding of how phage infection influences bacterial gene expression and, consequently, how bacterial responses to phage infection affect the assembly of polymicrobial communities. Using parallel genomic approaches, we have discovered novel transcriptional regulators and metabolic genes that influence phage infection. The integration of whole-genome transcriptomic profiling during phage infection has revealed the differential regulation of genes important for group behaviors and polymicrobial interactions. Our work suggests that therapeutic phages could more broadly influence bacterial community composition outside their intended host targets.

## INTRODUCTION

Enterococcus faecalis is a member of the healthy human intestinal microbiota (1). E. faecalis is also a pathobiont that rapidly outgrows upon antibiotic-mediated intestinal dysbiosis to cause disease. E. faecalis is associated with nosocomial sepsis, endocarditis, surgical-site, urinary tract, and mixed bacterial infections (2, 3). Since the 1980s, enterococci have been evolving extensive drug resistance, including resistance to vancomycin and “last-line-of-defense” antibiotics (4–10). In addition, E. faecalis can disseminate antibiotic resistance traits to diverse bacteria, including other clinically relevant pathogens (11–17). There is an urgent need for new therapeutics that target drug-resistant enterococci.

Bacteriophages (phages) are viruses that infect bacteria. Phages are being considered for the treatment of multidrug-resistant (MDR) bacterial infections, including enterococcal infections. Recent studies have demonstrated the potential for phage-based therapies against systemic and biofilm-associated enterococcal infections (18–22). The decolonization of intestinal MDR E. faecalis may be achieved through the action of phage predation which selects for cell wall variants that are rendered sensitive to antibiotic therapy (23). However, a potential barrier to the widespread use of phage therapy against E. faecalis is the development of phage resistance. To confront this issue, we must understand the molecular mechanisms used by phages to infect E. faecalis and how E. faecalis overcomes phage infection to become resistant. Only then can this biology be exploited to develop phage therapies that mitigate the risk of developing phage resistance.

The study of phage-bacterium interactions has provided key insights into phage infection that could lead to the development of novel antibacterial therapies. Phages replicate in bacteria by hijacking the host cellular machinery to produce phage progeny. To exploit host cell resources, many phages encode auxiliary proteins which are not directly involved in phage genome replication or particle assembly but can modulate bacterial physiology to favor phage propagation (24, 25). The characterization of phage auxiliary proteins may yield tools for curtailing bacterial infections. Additionally, the discovery of phage-modulated host pathways could reveal potential therapeutic targets. Our understanding of bacterial cellular responses during phage infection is limited to transcriptomic analyses in Gram-negative bacteria, whereas Gram-positive genera are understudied (26–31). Therefore, to fill this gap and further define the molecular underpinnings of enterococcal-phage interactions, we have taken a global genomics approach to identify enterococcal factors critical for productive infection by the lytic phage VPE25 (32). To identify bacterial genes essential for VPE25 infection, we screened a low-complexity transposon (Tn)-mutant library of E. faecalis OG1RF for phage resistance (33). In addition to the VPE25 receptor (32), transposon sequencing revealed novel E. faecalis genes necessary for phage adsorption and optimum intracellular phage DNA replication and transcription. To gain deeper insights into the physiological response of E. faecalis during phage infection, we used temporal transcriptomics of a VPE25 infection cycle. Transcriptomics revealed that VPE25 infection altered the expression of diverse genes involved in protein translation, metabolism, bacterial community sensing, virulence, and biofilm formation. Our work shows that E. faecalis reprograms transcription toward stress adaptation in response to phage infection. This suggests that phages may impact the behavior of bacteria in polymicrobial communities, including bystanders that are not the intended targets of phage therapy.

## RESULTS

### Transposon sequencing identifies novel genes involved in phage infection of E. faecalis.

**FIG 1:**
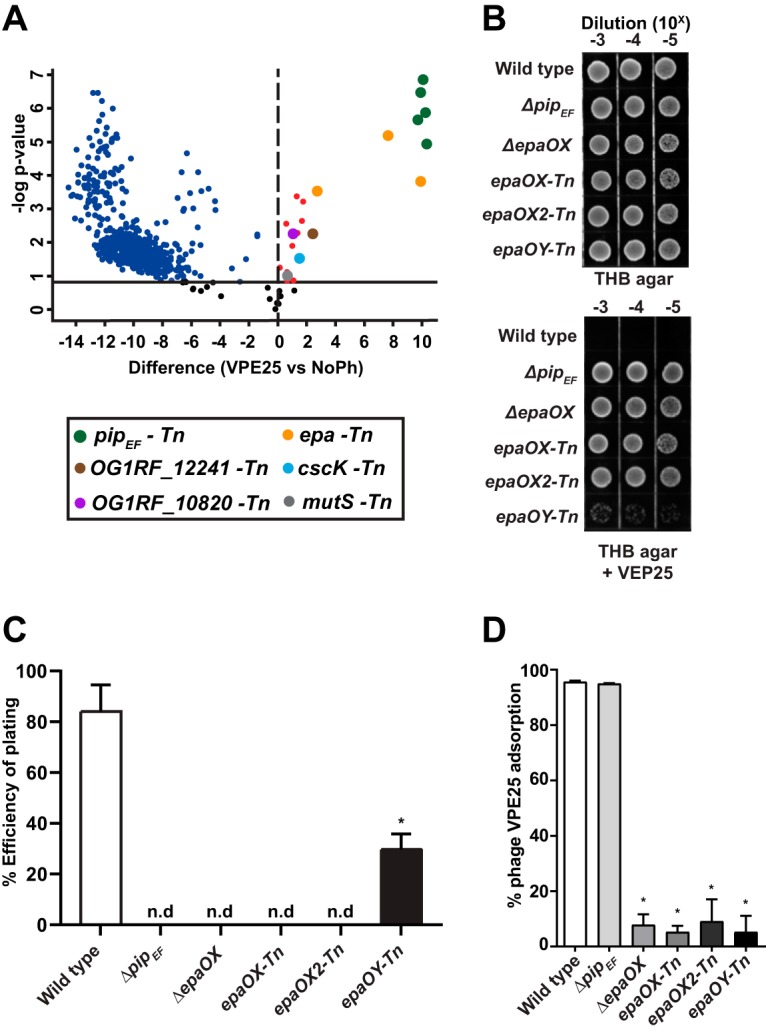
Transposon mutant library screening reveals E. faecalis genes important for productive phage infection. (A) Volcano plot demonstrating that phage challenge alters the abundance of select mutants from an E. faecalis OG1RF Tn library pool compared with phage VPE25-naive E. faecalis controls (false discovery rate, 0.05). Phage resistant/tolerant mutants of interest that are enriched upon phage exposure are highlighted as follows: *pip_EF_*-Tn (green), *epa*-Tn (yellow), *mutS*-Tn (gray), OG1RF_10820-Tn (purple), cscK-Tn (light blue), and OG1RF_12241-Tn (brown). (B) Phage VPE25 resistance phenotypes of an isogenic *epa* deletion strain or *epa*-specific Tn mutants serially diluted onto THB agar plates with or without 5 × 10^6^ PFU/ml of VPE25. (C) Infection efficiency of VPE25 is reduced in the *Δpip_EF_* and *epa* mutants compared with the wild type (n.d. indicates no detected plaques). (D) VPE25 efficiently adsorbs to wild-type E. faecalis OG1RF and an isogenic *Δpip_EF_* deletion strain but not to the various *epa* mutants.

To identify genetic determinants that confer phage resistance in E. faecalis, an E. faecalis OG1RF transposon library consisting of 6,829 unique mutants was screened by sequence-defined *mariner* technology transposon sequencing (SMarT Tn-Seq) (33). A total of 10^7^ CFUs of a logarithmically growing E. faecalis Tn-Seq library pool was plated on solid medium in the absence or presence of phage VPE25 at a multiplicity of infection (MOI) of 0.1. Cells from the input library prior to plating and cells plated onto plates containing no phage were used as controls. Tn insertions in E. faecalis genomic DNA were sequenced as described by Dale et al. (33). Sequencing reads were mapped to the E. faecalis OG1RF genome to identify bacterial mutants with altered phage sensitivity. The relative abundance of 22 E. faecalis mutants was enriched (adjusted *P* value of <0.05; log_2_ fold change, >0) in the presence of VPE25 relative to cultures that lacked phage and the input library (see Table S1 in the supplemental material; Fig. 1A). Five of the 22 phage-resistant enriched mutants harbored Tn insertions in OG1RF_10588 (Table S1; Fig. 1A), which was previously identified to encode the VPE25 receptor Phage Infection Protein of E. faecalis (Pip_EF_) (32). This indicates that the Tn mutant library is an appropriate tool for the discovery of genes involved in phage infection of E. faecalis.

10.1128/mBio.03120-19.8 TABLE S1 Differentially abundant transposon mutants during VPE25 selection of the E. faecalis OG1RF pooled Tn library. Download Table S1, XLSX file, 0.1 MB. Copyright © 2020 Chatterjee et al. 2020 Chatterjee et al. This content is distributed under the terms of the Creative Commons Attribution 4.0 International license.

To gain further insight into the genetic factors that influence E. faecalis susceptibility to VPE25, we analyzed several Tn mutants, including OG1RF_10820 (*lytR*), OG1RF_10951 (*cscK*), OG1RF_12241 (*lysR*), OG1RF_12435, and three enterococcal polysaccharide antigen (Epa)-associated genes, OG1RF_11715 (*epaOX*), OG1RF_11714, and OG1RF_11710. (Table S1; Fig. 1A). *epaOX* and OG1RF_11714 are both annotated as glycosyltransferases. We renamed OG1RF_11714 *epaOX2* as it resides downstream of *epaOX*. We have designated OG1RF_11710 as *epaOY* based on its homology to *epaY* (EF2169) in E. faecalis V583.

The *epa* genes encode proteins involved in the formation of a cell surface-associated rhamnose-containing polysaccharide (34). We and others have previously demonstrated that enterococcal phages unrelated to VPE25 utilize Epa to adsorb and infect E. faecalis (23, 35–37). Initial work from our group showed that mutation of the VPE25 receptor Pip_EF_ prevented VPE25 DNA entry into E. faecalis V583; yet, phages could still adsorb to receptor mutants (32), suggesting that the factors that promote phage infection via surface adsorption remained to be identified. Here, we show that either an in-frame deletion of *epaOX* (38) or Tn insertions in *epaOX*, *epaOX2*, and *epaOY* confer phage resistance to VPE25, similar to the *pip_EF_* receptor mutant (Fig. 1B). Mutations in *pip_EF_*_,_ *epaOX*, and *epaOX2* abolished VPE25 infection, while disruption of *epaOY* significantly reduced phage susceptibility, as determined by efficiency of plating assays (Fig. 1C). To assess the role of Epa during VPE25 infection, we investigated the ability of VPE25 to adsorb to wild-type E. faecalis, the *epaOX* deletion mutant, or the three *epa* Tn insertion mutants. Both the wild-type and the *pip_EF_* mutant strain adsorbed significantly larger amounts of VPE25 than the *epa* mutants (Fig. 1D). Together, these data indicate that *epa*-derived cell wall modifications contribute to VPE25 infection by promoting surface adsorption. This is consistent with previous observations in other lactic acid bacteria showing phage infection is a two-step process; first, phages must reversibly bind to a cell wall polysaccharide, followed by the committed initiation of DNA ejection into the cell (39–41).

**FIG 2:**
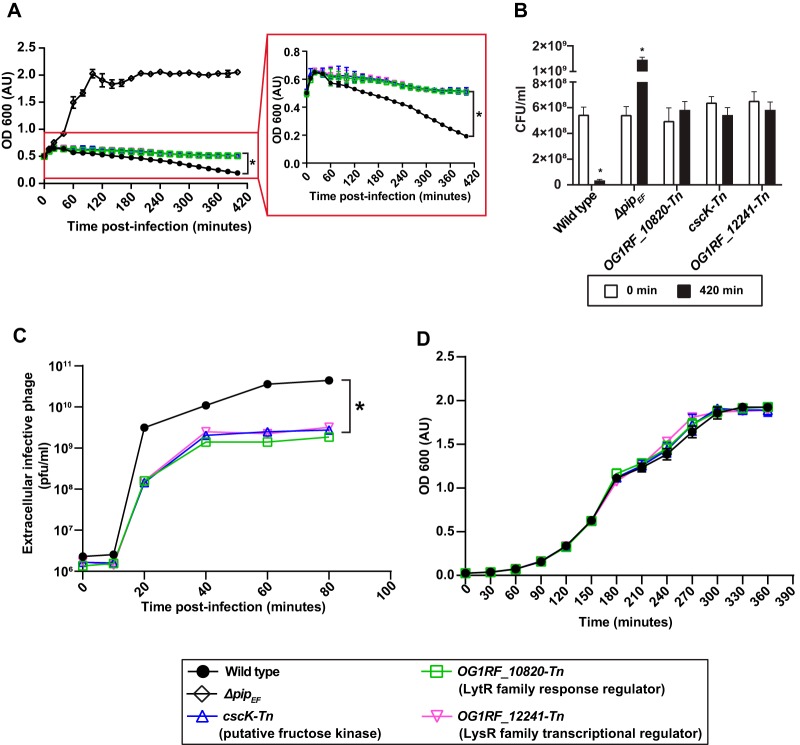
VPE25-mediated killing is halted and phage production is reduced during infection of OG1RF_10820-Tn (*lytR*), cscK-Tn, and OG1RF_12241-Tn (*lysR*) transposon mutants. (A) VPE25 killing curves, (B) CFUs per ml at the beginning (0 min) and endpoint (420 min) of phage infection, and (C) VPE25 particle production kinetics using the indicated E. faecalis transposon mutant strains compared with the wild-type or *Δpip_EF_* deletion strains. The inset highlights the delayed lysis phenotype of the transposon mutants relative to wild type. (D) Growth curves of all the strains in the absence of VPE25. Data show three independent experiments combined and presented as the mean with standard deviation. *P < 0.0001 by two-way analysis of variance (ANOVA).

In addition to *epa* genes, several Tn mutants whose roles during phage infection were unknown were enriched on VPE25-containing agar compared with uninfected controls (Table S1; Fig. 1A), and this included OG1RF_10820-Tn, *cscK*-Tn, and OG1RF_12241-Tn. OG1RF_10820 encodes a putative LytR response regulator. Orthologs of this protein control multiple cellular processes, including virulence, extracellular polysaccharide biosynthesis, quorum sensing, competence, and bacteriocin production (42). OG1RF_12241 is a homolog of the *hypR* (EF2958) gene of E. faecalis strain JH2-2 and encodes a LysR family transcriptional regulator. HypR regulates oxidative stress through the *ahpCF* (alkyl hydroperoxide reductase) operon conferring increased survival in mouse peritoneal macrophages (43, 44). Finally, CscK is a fructose kinase that converts fructose to fructose-6-phosphate for entry into glycolysis (45). Considering that these genes had never previously been shown to be associated with phage infection, we asked how Tn disruption of these genes influenced phage sensitivity using a time course phage infection assay. In the presence of phages, the optical density of the Tn mutants OG1RF_10820-Tn (*lytR*), *cscK-*Tn, and OG1RF_12241-Tn (*lysR*) was constant over time, whereas the growth of the wild type and the *pip_EF_* receptor mutant declined or increased over the course of infection, respectively (Fig. 2A and B). Complementation of the Tn mutants with wild-type alleles restored phage susceptibility without altering their growth in the absence of phages (see Fig. S1A, B, and C in the supplemental material). To further investigate this phage tolerance phenotype, we asked whether these mutants harbored a defect in phage production. Assessment of the number of phage particles produced during infection showed that OG1RF_10820-Tn (*lytR*), *cscK*-Tn, and OG1RF_12241-Tn (*lysR*) strains produced 10-fold lower phage particles than the wild-type strain (Fig. 2C), indicating that the Tn mutants have a defect in phage burst size. In the absence of phage, wild-type, *Δpip_EF_*, OG1RF_10820-Tn (*lytR*), *cscK*-Tn, and OG1RF_12241-Tn (*lysR*) strains showed similar growth kinetics (Fig. 2D), indicating that these mutations do not impair growth in laboratory media. These data show that deficiencies in transcriptional signaling and metabolism have a strong impact on lytic phage production in E. faecalis.

10.1128/mBio.03120-19.2 FIG S1 Complementation restores phage sensitivity in OG1RF_10820 (*lytR*), *cscK*, and OG1RF_12241 Tn (*lysR*) mutants. (A and B) Introduction of the wild-type allele but not the empty plasmid sensitizes E. faecalis Tn mutants to phage VPE25 infection. (C) Growth in the absence of VPE25 remains unaltered irrespective of the presence of the empty or complementation plasmid. (E, empty vector; C, complemented). Data represent the average of three replicates ± the standard deviation. *P < 0.0001 by unpaired Student’s t-test. Download FIG S1, PDF file, 0.6 MB. Copyright © 2020 Chatterjee et al. 2020 Chatterjee et al. This content is distributed under the terms of the Creative Commons Attribution 4.0 International license.

Since we observed that OG1RF_10820-Tn, *cscK*-Tn, and OG1RF_12241-Tn (*lysR*) mutants had a reduced phage burst, releasing fewer viral particles relative to the wild type, we queried the status of viral transcription and replication in these mutants. Whole-genome transcriptomic analysis of phage-infected E. faecalis OG1RF cells indicated that the VPE25 genes *orf76*, *orf111*, and *orf106* are highly expressed throughout the course of phage infection (see Table S2A in the supplemental material). However, the transcripts of these genes were less abundant in the Tn mutants OG1RF_10820-Tn (*lytR*), *cscK*-Tn, and OG1RF_12241-Tn (*lysR*) (see Fig. S2A in the supplemental material). Additionally, phage DNA replication is delayed in these Tn insertion mutants, as judged by significantly lower copy numbers of phage DNA relative to the wild-type strain (Fig. S2B). These data indicate that OG1RF_10820-Tn (*lytR*), *cscK*-Tn, and OG1RF_12241-Tn (*lysR*) mutants restrict VPE25 DNA replication by increasing the phage latent period, resulting in reduced phage particle production.

10.1128/mBio.03120-19.3 FIG S2 Dampened viral gene expression and DNA replication aids in OG1RF_10820-Tn (*lytR*), *cscK*-Tn, and OG1RF_12241-Tn (*lysR*) mutant tolerance to VPE25 infection. (A) Effect of various Tn insertion mutations on VPE25 mRNA levels. The data are shown as the fold change of normalized mRNA compared with the wild-type samples at various time points postinfection. (B) VPE25 DNA copy number calculated from an *orf76* standard curve. Data are represented as an average of three replicates ± the standard deviation. *P < 0.00001 by unpaired Student’s t-test. Download FIG S2, PDF file, 0.5 MB. Copyright © 2020 Chatterjee et al. 2020 Chatterjee et al. This content is distributed under the terms of the Creative Commons Attribution 4.0 International license.

10.1128/mBio.03120-19.9 TABLE S2 (A) Differential expression ratio of VPE25 genes during E. faecalis OG1RF infection relative to the start of infection. (B) Differential expression ratio of E. faecalis OG1RF genes during VPE25 infection relative to the untreated cultures. (C) GO and KEGG annotations of enterococcal genes downregulated throughout the course of VPE25 infection. (D) GO and KEGG annotations of enterococcal genes upregulated during VPE25 infection. Download Table S2, XLSX file, 0.2 MB. Copyright © 2020 Chatterjee et al. 2020 Chatterjee et al. This content is distributed under the terms of the Creative Commons Attribution 4.0 International license.

### Mutation of *OG1RF_10820* (*lytR*) alters *epa* variable gene expression, negatively impacting phage infection.

**FIG 3:**
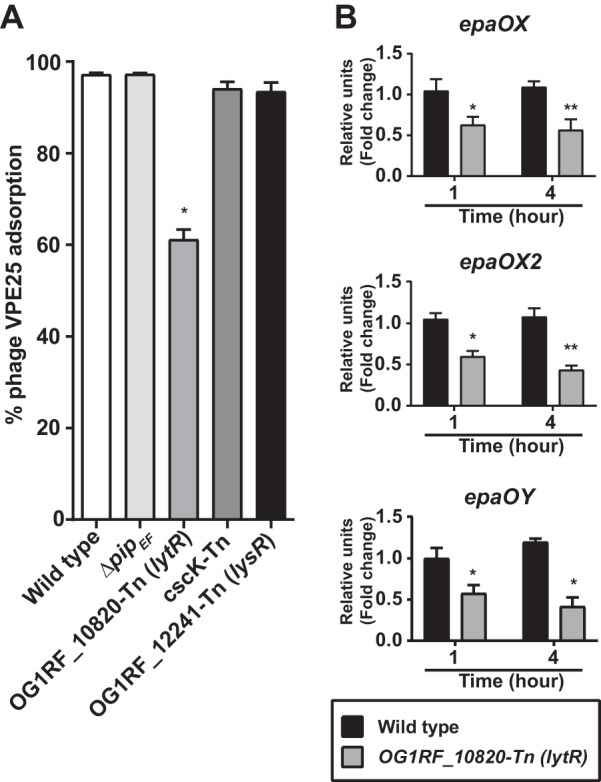
Mutation of a *lytR* homolog downregulates the expression of *epa* variable genes, leading to decreased VPE25 adsorption. (A) Phage adsorption assay showing that the OG1RF_10820-Tn (*lytR*) mutant strain is defective for VPE25 attachment relative to the wild-type strain. (B) Disruption of *OG1RF_10820* (*lytR*) leads to reduced expression of three *epa* variable genes, namely, *epaOX* (top), *epaOX2* (middle), and *epaOY* (bottom). The data are represented as the fold change of normalized mRNA compared with wild type during both logarithmic (1 h) and stationary-phase (4 h) growth. The data show the average of three biological replicates ± the standard deviation. *P < 0.01, **P < 0.001 by unpaired Student’s *t* test.

To further assess the roles of the *OG1RF_10820* (*lytR*), *cscK*, and *OG1RF_12241* (*lysR*) genes during VPE25 infection, we performed phage adsorption assays with these strains. OG1RF_10820-Tn (*lytR*), which harbors a Tn-disrupted *lytR* response regulator gene, adsorbed 40% less phage than the wild-type control (Fig. 3A). Of the three Tn mutants, this was the only mutant that adsorbed less phage than the wild-type control. LytR type response regulators have been implicated in the biosynthesis of extracellular polysaccharides, including alginate biosynthesis in Pseudomonas aeruginosa (42, 46, 47). Since phage adsorption of E. faecalis is facilitated by Epa, we measured *epa* gene expression in the OG1RF_10820-Tn (*lytR*) background. The *epa* locus consists of core genes (*epaA* to *epaR*) that are conserved in E. faecalis, followed by a group of strain-specific variable genes that reside downstream of the core genes (37, 48). The expression of *epa* variable genes *epaOX*, *epaOX2*, and *epaOY* were reduced in the absence of OG1RF_10820 (*lytR*) during logarithmic and stationary-phase growth (Fig. 3B). In contrast, OG1RF_10820 (*lytR*) disruption did not alter the expression of core *epa* genes (see Fig. S3 in the supplemental material). Collectively, these results indicate that mutation of the *lytR* homolog hinders optimum binding of VPE25 by downregulating *epa* variable genes, thereby modifying the polysaccharide decoration of the core Epa structure (49).

10.1128/mBio.03120-19.4 FIG S3 The *lytR* gene does not influence the expression of *epa* core genes. Quantitative PCR demonstrates equivalent mRNA levels of the *epa* genes *epaA*, *epaE*, and *epaR* in wild type and the OG1RF_10820-Tn (*lytR*) E. faecalis strains. The data are expressed as fold change of normalized mRNA compared with the wild type at different time points and represent the average of three biological replicates ± the standard deviation. Download FIG S3, PDF file, 0.4 MB. Copyright © 2020 Chatterjee et al. 2020 Chatterjee et al. This content is distributed under the terms of the Creative Commons Attribution 4.0 International license.

### Hypermutator strains defective in mismatch repair facilitate the acquisition of phage resistance in E. faecalis.

**FIG 4:**
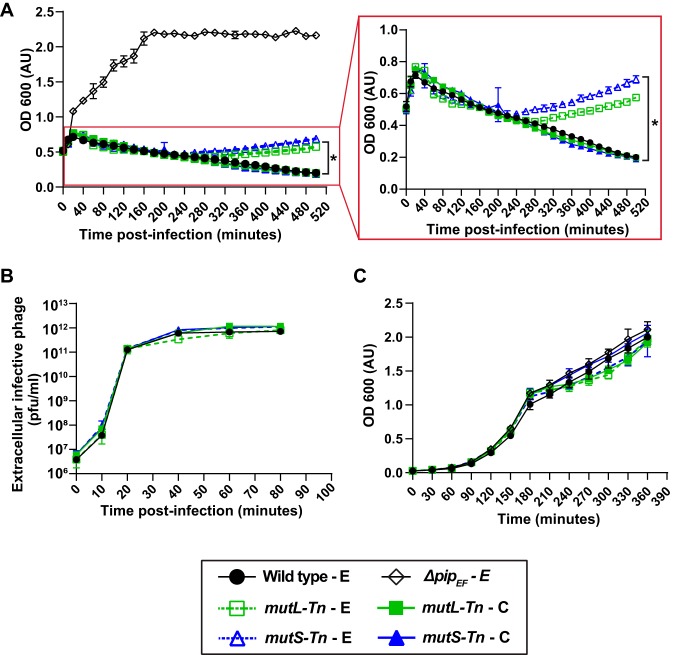
Emergence of phage resistance in E. faecalis *mutL-Tn* and *mutS-Tn* strain backgrounds. (A) Culture density of E. faecalis *mutL*-Tn-E and *mutS*-Tn-E strains declined similar to the wild-type E strain following VPE25 infection. However, VPE25 resistance gradually emerged in the *mutL*-Tn-E and *mutS*-Tn-E strain backgrounds, as indicated by an increase in cell density following VPE25 infection. (B) *mutL*-Tn-E, *mutS*-Tn-E, and wild-type E strains release equivalent number of phages (PFU/ml) during the course of infection, and (C) grow similarly in the absence of phage. Data show the average of three biological replicates ± the standard deviation. (E, empty vector; C, complemented). *P < 0.0001 by two-way analysis of variance (ANOVA).

Transposon mutant OG1RF_12435-Tn, with an insertion in the DNA mismatch repair (MMR) gene *mutS*, was significantly overrepresented during VPE25 infection (Table S1; Fig. 1A). The MMR genes *mutS* and *mutL* correct replication-associated mismatch mutations (50). We discovered that VPE25-mediated lysis of the *mutS*-Tn-E (OG1RF_12435-Tn carrying the empty plasmid pAT28) and *mutL*-Tn-E (OG1RF_12434-Tn carrying the empty plasmid pAT28) mutants closely resembled wild-type lysis kinetics for ∼4 hours postinfection and released similar numbers of phage particles (Fig. 4A and B). However, these mutator strains eventually started to recover and escape infection, suggesting that the mutator phenotype gives rise to phage resistance (Fig. 4A). Introduction of the wild-type *mutS* and *mutL* genes cloned into plasmid pAT28 (*mutS*-Tn-C and *mutL*-Tn-C) restored the wild-type phage susceptibility phenotype (Fig. 4A). In the absence of phage, the wild-type, *mutL*-Tn, and *mutS*-Tn strains grew similarly, suggesting that hypermutator strains do not harbor growth defects *in vitro* (Fig. 4C).

To confirm that the *mutS*-Tn and *mutL*-Tn strains accumulate phage-resistant isolates during phage exposure, we performed phage infection assays using colonies of *mutS*-Tn and *mutL*-Tn grown overnight on agar plates in the absence and presence of VPE25. Consistent with our previous data, all *mutS*-Tn and *mutL*-Tn mutant colonies selected from agar plates lacking phage were initially phage sensitive, and over time became phage resistant (see Fig. S4A and C in the supplemental material). In contrast, the *mutL-*Tn and *mutS-*Tn colonies acquired from the phage-containing plates were phage resistant and had similar growth kinetics to the *pip_EF_* receptor mutant in the presence of VPE25 (Fig. S4A and C). All isolates grew similarly in the absence of phage (Fig. S4B and D). To gain insight into the basis of acquired phage resistance in the mismatch repair mutant backgrounds, we compared the phage adsorption profiles of the different strains. The *mutL*-Tn and *mutS*-Tn mutants that were not preexposed to VPE25 adsorbed phage at 70% to 80% efficiency, whereas *mutL-*Tn and *mutS-*Tn colonies chosen from phage-containing agar plates displayed a severe phage adsorption defect (Fig. S4E and F). Our data show that phage treatment leads to the selection and growth of phage adsorption-deficient isolates from *mutL*-Tn and *mutS*-Tn mutator cultures, most likely through mutations in *epa* variable genes. This also suggests that *epa* variable genes may be a hot spot for mutation in E. faecalis.

10.1128/mBio.03120-19.5 FIG S4 Mutator strains facilitate the acquisition of VPE25 resistance. Growth of wild type, *Δpip*, parent *mutL-Tn*, *mutL-Tn* colonies selected from THB plate without phage (mutL-Tn-NPh_C1 to C4 and mutS-Tn-NPh_C1 to C4), and those selected from VPE25 (5 × 10^6^ PFU/ml) containing THB plates (mutL-Tn-Ph_C1 to C4 and mutS-Tn_C1 to C4) are shown in the presence (A and C) and in the absence (B and D) of VPE25. The mutL-Tn and mutS-Tn colonies preexposed to phage behave similar to phage-resistant *Δpip*, whereas hypermutator colonies without previous VPE25 challenge gained phage resistance during the course of infection. (E and F) All the mutL-Tn-Ph_C1 to C4 and mutS-Tn-Ph_C1 to C4 have a defective VPE25 adsorption profile, while colonies selected from no-phage plates are able to adsorb ∼70% to 80% of the phages in the assay. Download FIG S4, PDF file, 1.1 MB. Copyright © 2020 Chatterjee et al. 2020 Chatterjee et al. This content is distributed under the terms of the Creative Commons Attribution 4.0 International license.

### VPE25 infection drives global gene expression changes in E. faecalis.

**FIG 5:**
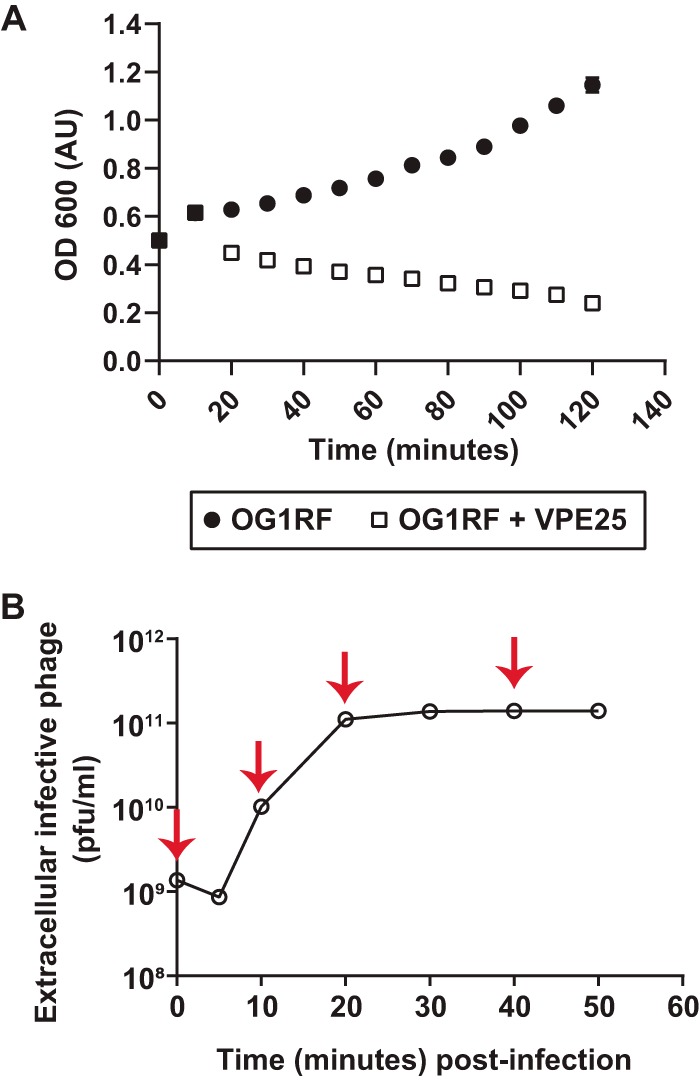
Bacterial growth curve and one-step phage burst kinetics. (A) Optical density of E. faecalis OG1RF cultures in the presence and absence of VPE25 infection (MOI, 10). (B) One-step VPE25 growth curve during the infection of E. faecalis OG1RF. The red arrows indicate the time points selected for transcriptome analysis. Data from three independent experiments are combined and presented as the mean with standard deviation.

To study temporal changes in E. faecalis gene expression during phage infection, we infected logarithmically growing E. faecalis with VPE25 at an MOI of 10. The cell density of infected E. faecalis cultures was comparable to the uninfected control cultures during the first 10 min of infection (Fig. 5A). Between 10 and 20 min postinfection, the VPE25 burst size increased as the cell density of the infected culture declined (Fig. 5A and B). VPE25 particle numbers plateaued 30 min postinfection, and there was no significant increase in phage output between 30 and 50 minutes of infection (Fig. 5B).

**FIG 6:**
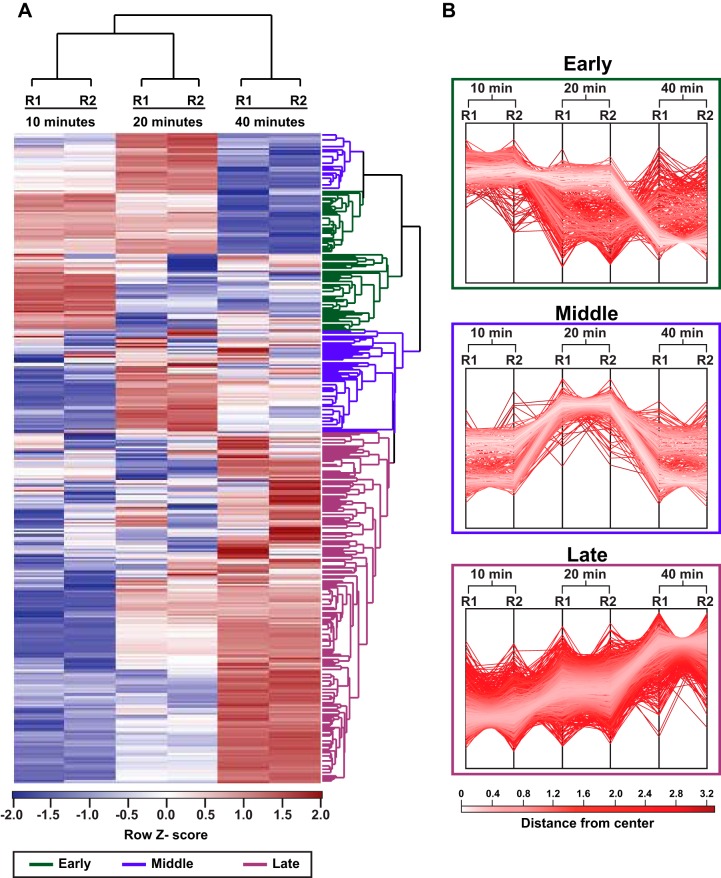
Global transcriptomic profile of E. faecalis OG1RF in response to VPE25. (A) Hierarchical clustering of differentially expressed E. faecalis transcripts at 10, 20, and 40 min post-VPE25 infection compared with uninfected controls from each time point. R1 and R2 designate two independent biological replicates. The transcripts broadly cluster into early (green), middle (blue), and late (magenta) expressed genes. (B) The profile plots of the early (top), middle (central), and late (bottom) clusters are shown. Each line indicates a gene within a cluster, and the color intensity is calculated based on the distance from the center value in that cluster.

To investigate the transcriptional response of E. faecalis during VPE25 infection, we collected cells at several time points during distinct phases of the VPE25 infection cycle and performed RNA sequencing (RNA-Seq). Samples were collected at 10, 20, and 40 minutes postinfection, representing the early, middle, and late phase of the VPE25 infection cycle, respectively (Fig. 5A and B). Although phage replication peaks at 20 minutes, there is a modest reduction of the optical density at 600 nm (OD_600_) between 20 and 40 minutes, indicating that some cells in the culture may be more resistant to phage infection. Hierarchical clustering of differentially expressed E. faecalis genes during VPE25 infection compared with uninfected controls revealed unique gene expression patterns at each time point (Fig. 6A and B; Table S2B). Gene expression patterns grouped into three distinct clusters that correlated with the early, middle, and late stages of phage infection (Fig. 6A and B). Phages rely on host cell resources for the generation of viral progeny. Gene Ontology (GO) and KEGG enrichment analysis showed that phage infection influenced numerous E. faecalis metabolic pathways, including amino acid, carbohydrate, and nucleic acid metabolism (Table S2B).

Approximately 54% of the E. faecalis genome was differentially expressed (*P < *0.05) relative to uninfected controls, with 692 downregulated and 731 upregulated genes over the course of phage infection (see Fig. S5A and C in the supplemental material). A total of 37 of the 692 downregulated genes were repressed throughout the course of phage infection and are broadly categorized as ribosome biogenesis and bacterial translation genes (Fig. S5B; Table S2C), indicating that VPE25 modulates host protein biogenesis to prevent bacterial growth and promote viral replication. In contrast, expression of 110 genes belonging to DNA repair pathways, amino acyl-tRNA biosynthesis, and carbohydrate metabolism were significantly upregulated throughout the phage infection cycle (Fig. S5D; Table S2D). The induction of DNA stress response genes suggests that E. faecalis cells activate DNA defense mechanisms to counteract phage-driven DNA damage.

10.1128/mBio.03120-19.6 FIG S5 VPE25 modulation of E. faecalis genes during infection. (A) Euler diagram representing the number of host transcripts with reduced abundance during phage infection. (B) Among the 37 genes that are downregulated throughout the entire phage infection cycle, a large percent belong to ribosome biogenesis and translation. (C) Phage-induced expression of multiple host transcripts include those involved in DNA replication and repair (D), tRNA biosynthesis, and carbohydrate metabolism. Download FIG S5, PDF file, 0.4 MB. Copyright © 2020 Chatterjee et al. 2020 Chatterjee et al. This content is distributed under the terms of the Creative Commons Attribution 4.0 International license.

Next, we assessed the transcriptome of VPE25 during infection. The VPE25 genome encodes 132 open reading frames (ORFs). We detected the differential expression of these ORFs by comparing the average read counts of individual genes at 10, 20, and 40 min relative to the start of infection (0 min). Hierarchical clustering grouped differentially expressed genes into early and late genes based on their distinct temporal expression patterns. The transcripts of 78 early genes, including those predicted to be involved in nucleotide biosynthesis and replication, accumulated during the first 10 min of infection (see Fig. S6A in the supplemental material; Table S2A). In contrast, late genes encoding phage structural components, DNA packaging, and host cell lysis were induced by 40 min of infection (Fig. S6A; Table S2A). Approximately 90 genes (68% of the VPE25 genome) annotated as hypothetical in the VPE25 genome were expressed during the early or late phase of infection (Fig. S6A; Table S2A), indicating that the majority of actively transcribed genes during VPE25 infection have no known function.

10.1128/mBio.03120-19.7 FIG S6 Transcriptomic profiling reveals alterations to phage and bacterial gene expression patterns throughout the course of infection. (A) Hierarchical clustering of differentially expressed VPE25 transcripts after 10, 20, and 40 minutes relative to 0 minutes post-viral infection. One and 2 designate individual biological replicates. The transcripts are broadly classified into early and late gene clusters depicted in purple and green, respectively. (B) Volcano plots demonstrate changes in the gene expression pattern of E. faecalis OG1RF during VPE25 infection. Volcano plots highlight differentially expressed genes in the bacteria at 10 min (top), 20 min (middle), and 40 min (bottom) post-VPE25 infection. Downregulated genes are shown in blue and upregulated genes are in red (fold change, >2; *P < *0.01). (C to E) Successful phage infection modulates *fsr* quorum sensing and T7SS genes. VPE25 administration at an MOI of 1 or 10 (D) downregulates *fsr* quorum sensing and upregulates T7SS in E. faecalis OG1RF but not in *Δpip_EF_* (MOI, 10) (D). (E) The Pip_EF_ independent phage NPV-1 was used to query the expression of select *fsr*-associated genes (*fsrBD* and *sprE*) and type VII secretion genes (*OG1RF_11100*, *OG1RF_11101*, *OG1RF_11104*, *OG1RF_11105*, and *OG1RF_11115*) by qPCR 1 h after NPV-1 infection of E. faecalis OG1RF. The data are expressed as fold change of normalized mRNA compared with the uninfected controls and represent an average of three biological replicates ± the standard deviation. *P < 0.0001 and **P < 0.00001 by unpaired Student’s t-test. Download FIG S6, PDF file, 1.7 MB. Copyright © 2020 Chatterjee et al. 2020 Chatterjee et al. This content is distributed under the terms of the Creative Commons Attribution 4.0 International license.

### Phage infection modulates E. faecalis genes involved in group interactions.

Our transcriptomic data indicate that VPE25 infection causes a shift in the expression pattern of genes involved in pathways unrelated or peripheral to host metabolism and macromolecule biosynthesis (Fig. S5B and D). Most notably, we observed that phage infection led to a significant reduction in the expression of *fsr* quorum sensing genes and in the induction of type VII secretion system (T7SS) genes (Fig. S6B).

**FIG 7:**
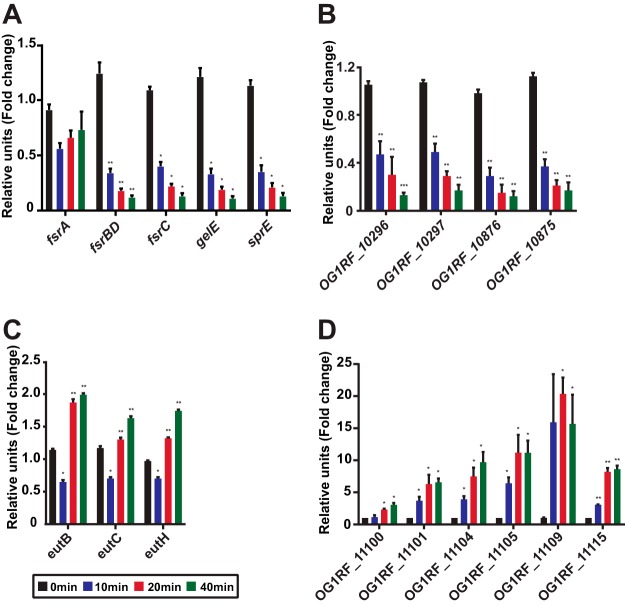
Quantitative PCR confirms altered expression of bacterial quorum sensing and T7SS genes during VPE25 infection. mRNA transcript levels of quorum sensing regulon genes, including *fsr* regulatory genes (A), *fsr*-induced genes (B), and *fsr*-repressed genes (C) are differentially expressed during VPE25 infection. (D) Progression of the lytic cycle induces the expression of T7SS genes. Expression is the fold change relative to untreated samples at the same time points. Data represent the average of three replicates ± the standard deviation. *P < 0.01 to 0.0001 and **P < 0.00001 by unpaired Student’s *t* test.

The E. faecalis *fsr* quorum sensing system is critical for virulence and biofilm formation in different animal models, including mouse models of endocarditis and peritonitis (51–55). The *fsr* quorum sensing system is comprised of the *fsrA*, *fsrBD*, and *fsrC* genes (56–58). *fsrA* encodes a response regulator that is constitutively expressed. *fsrBD* and *fsrC* encode the accessory protein, pheromone peptide, and the membrane histidine kinase required for functional quorum sensing-regulated gene expression in E. faecalis. Quantitative real-time PCR (qPCR) confirmed that *fsrBD* and *fsrC* are repressed throughout VPE25 infection, with the strength of repression increasing over time (Fig. 7A). There was a negligible impact on *fsrA* mRNA levels (Fig. 7A), consistent with its behavior as a constitutively expressed gene. qPCR analysis revealed several Fsr-controlled genes to be differentially expressed during phage infection. Fsr-dependent virulence factors, including *gelE*, *sprE*, *OG1RF_10875* (*EF1097*), and *OG1RF_10876* (*EF1097b*) genes (59), were all significantly downregulated during phage infection relative to uninfected controls (Fig. 7A and B). The *fsr* regulon is also an activator and repressor of several metabolic pathways (60). Our data demonstrate that the levels of Fsr-activated genes involved in the phosphotransferase sugar transport system (OG1RF_10296 and OG1RF_10297) are reduced, whereas negatively regulated genes in the *fsr* regulon, such as *eutB*, *eutC*, and *eutH* genes involved in ethanolamine utilization, are derepressed during VPE25 infection (Fig. 7B and C). These data suggest that VPE25 attenuates the FsrABDC system, consequently impacting E. faecalis virulence and metabolism.

Divergent forms of the T7SS (also known as the Ess/Esx system) are widely distributed in Gram-positive bacteria, and the T7SS has been extensively studied in the actinobacterium Mycobacterium tuberculosis (61–66). To date, enterococcal T7SS loci remain uncharacterized. Consistent with our transcriptomic data (Fig. S6B), we observed that phage infection induces genes in the E. faecalis T7SS locus, including *OG1RF_11100* (*esxA*), *OG1RF_11101* (*essA*), *OG1RF_11104* (*essB*), *OG1RF_11105* (*essC1*), *OG1RF_11109*, and *OG1RF_11115* (*essC2*) (Fig. 7D). The *esxA* and *OG1RF_11109* genes encode potential WXG100 domain-containing effector and LXG-domain toxin proteins, respectively. The secretion of T7SS factors is dependent on the EssB transmembrane protein and FtsK/SpoIIIE ATPases encoded by the *essC1* and *essC2* genes. The T7SS in Gram-positive bacteria is involved in immune system activation, apoptosis of mammalian cells, bacterial cell development and lysis, DNA transfer, and bacterial interspecies interactions (62, 67–77).

Similar to other phage-bacterium interaction studies (26, 28–30, 78), we investigated the transcriptional response of E. faecalis during VPE25 infection using a high MOI (MOI, 10) to increase the probability of phage infection and minimize transcriptional noise from uninfected bacterial cells. However, a lower MOI (MOI, 1) is sufficient to alter the expression profile of at least a subset of quorum sensing and T7SS genes (Fig. S6C). Additionally, administration of VPE25 (MOI, 10) to the phage-resistant E. faecalis strain *Δpip_EF_* did not alter mRNA levels of *fsr* quorum sensing or T7SS genes (Fig. S6D), suggesting that successful phage infection is essential to modulate the expression of these bacterial genes. To investigate whether phage-mediated expression of *fsrABDC* and the T7SS genes are VPE25 specific, we examined the expression patterns of a subset of these genes in E. faecalis when infected with the Pip_EF_-independent lytic phage NPV-1, which shares little genetic similarity to phage VPE25 (2.4% pairwise nucleotide identity across its genome). mRNA levels of *fsrB* and *sprE* were reduced, whereas expression of the T7SS genes were elevated during NPV-1 infection (Fig. S6E), suggesting that phage-specific control of quorum sensing and T7SS expression are not restricted to VPE25 infection. Together, these findings indicate that phage infection of E. faecalis has the potential to influence bacterial adaptation in polymicrobial communities or during mixed bacterial species infections.

## DISCUSSION

Characterizing bacterial responses to phage infection is important for understanding how phages modulate bacterial physiology and will inform approaches toward effective phage therapies against MDR bacteria. Commonly used approaches to identify phage-resistant bacteria often yield information restricted to phage receptors and/or adsorption mechanisms. While useful, these approaches often overlook more subtle interactions that drive the efficiency of phage infection and phage particle biogenesis. Additionally, these approaches provide minimal information on how bacteria sense and respond to phage infection. These are important gaps in knowledge considering the heightened interest in utilizing phages as clinical therapeutics against difficult-to-treat bacterial infections. To begin to address these knowledge gaps we have studied the model Gram-positive commensal and opportunistic pathogen E. faecalis when infected with its cognate lytic phage VPE25. We have discovered several novel bacterial factors that are indispensable for efficient VPE25 infection of E. faecalis. In addition, we have uncovered key insights into the molecular events that are triggered in E. faecalis cells during phage infection. Importantly, our work shows that E. faecalis alters the expression of genes associated with environmental sensing and group interactions during phage infection. Such a response may have unexpected consequences in polymicrobial communities, and our work sets the stage for studying how phage therapies may impact nontarget bacteria in the microbiota.

Tn-Seq identified numerous E. faecalis genes that govern VPE25 susceptibility. Mutations in *epa* variable genes conferred VPE25 resistance by preventing phage adsorption, similar to other E. faecalis phages (23, 35–37). We discovered that phage infection of mismatch repair gene mutants results in the emergence of phage adsorption deficiencies; thus, the mismatch repair system likely fails to correct DNA damage of *epa* genes during phage infection. This suggests that *epa* genes may be a hot spot for mutation. Tn-Seq also enabled the discovery of the LytR-domain transcription factor encoded by OG1RF_10820 (*lytR*) as a regulator of *epa* variable locus gene expression (Fig. 3B). Considering an *epa* mutant strain of E. faecalis is defective in colonization and outgrowth during antibiotic selection in the intestine (23), we believe that future investigation of LytR-mediated regulation of *epa* and potentially other genomic loci could guide the development of effective therapeutics to control E. faecalis colonization and infections.

In this work, we have discovered bacterial metabolic and oxidative stress response genes that are important for phage infection. Decreased phage replication coupled with lower burst size of VPE25 in the fructose kinase mutant (*cscK*) suggests that VPE25 relies on host carbohydrate metabolism to support viral progeny formation. The reliance of VPE25 on host CscK for viral propagation is corroborated by RNA-Seq data showing broad induction of bacterial host carbohydrate metabolism genes during VPE25 infection. Furthermore, we observed that VPE25 lytic growth leads to a gradual rise in the OG1RF_12241 (*lysR*) transcripts which encode the LysR homolog HypR, a regulator of oxidative stress. Phage infection enhanced the transcript levels of three other oxidative stress response genes, including OG1RF_10348, OG1RF_10983, and OG1RF_11314 encoding superoxide dismutase (*sodA*), NADH peroxidase (*npr*), and catalase (*katA*), respectively (Table S2B). In Campylobacter jejuni, mutations in the LysR-regulated gene *ahpC*, as well as *sodB* and *katA*, resulted in reduced plaquing efficiency by the phage NCTC 12673 (27). We hypothesize that phage tolerance during hypersensitivity to oxidative stress could be detrimental to E. faecalis and targeting such pathways could be used to control E. faecalis colonization.

Our data indicate that putative T7SS genes are activated in response to phage infection. Although E. faecalis T7SS remains poorly characterized, the Staphylococcus aureus T7 system has been demonstrated to defend cells against neutrophil assault and enhance epithelial cell apoptosis and is critical for virulence (68, 69, 79, 80). Additionally, S. aureus T7SS maintains membrane homeostasis and is involved in the membrane stress response (69, 81). The finding that E. faecalis T7SS genes are induced in response to two different phages suggests phage-mediated membrane damage may lead to elevated T7SS gene expression. Finally, the S. aureus T7SS nuclease toxin EsaD contributes to interspecies competition through growth inhibition of rival strains lacking the EsaG antitoxin (77). The impact of S. aureus T7SS on rival strains and the presence of T7SS genes in environmental isolates (82, 83) suggest a pivotal role of this secretion system in shaping microbial communities.

In contrast to the T7SS, the bacterial population-associated quorum sensing *fsr* locus was repressed during VPE25 infection in E. faecalis. Gram-negative bacteria can escape phage invasion by quorum sensing-mediated downregulation of phage receptor expression or activation of clustered regularly interspaced short palindromic repeats (CRISPR)-Cas immunity (84–87). The *fsr* regulon does not include the receptor (*pip_EF_*), *epa* genes necessary for phage adsorption, or CRISPR-Cas. However, the *fsr* system does contribute to biofilm formation that could potentially deter phage infection (55, 88). VPE25 infection may attenuate *fsr*-mediated biofilm formation to favor the continued infection of neighboring planktonic cells. On the other hand, phage genomes have been shown to carry enzymes that degrade quorum sensing molecules or anti-CRISPR genes to evade host defense strategies (89, 90). Although such anti-host accessory genes are not evident in the VPE25 genome, it is possible that phage-carrying hypothetical genes influence E. faecalis quorum sensing and dictate molecular events that favor phage production.

Integration of global transcriptomics and transposon library screening of VPE25-infected E. faecalis has revealed new insights into our understanding of phage-host interactions in enterococci. Together, our results emphasize the importance of *epa* gene regulation, carbohydrate metabolism, and the oxidative stress response in successful phage predation. Furthermore, contributions of VPE25 on E. faecalis *fsr* and T7SS genes involved in inter- and intrabacterial interactions suggest that phage therapy could impact microbial community dynamics in patients undergoing treatment, and such an outcome should be considered for the development of phage-based therapeutics.

## MATERIALS AND METHODS

### Bacteria and bacteriophages.

All bacteria and phages used in this study are listed in Table S3 in the supplemental material. E. faecalis strains were grown with aeration on Todd-Hewitt broth (THB) or THB agar at 37°C. Escherichia coli was grown on Lennox L broth (LB) with aeration or on LB agar at 37°C. The following antibiotic concentrations were added to media for selection of E. coli or E. faecalis: 25 μg/ml fusidic acid, 50 μg/ml rifampin, 750 μg/ml spectinomycin, and 20 μg/ml chloramphenicol. Phage sensitivity assays were performed on THB agar supplemented with 10 mM MgSO_4_. Phages used for all experiments were purified by cesium chloride gradient separation as described previously (32).

10.1128/mBio.03120-19.10 TABLE S3 Bacterial strains, phages, plasmids, and primers used in this study. Download Table S3, PDF file, 0.2 MB. Copyright © 2020 Chatterjee et al. 2020 Chatterjee et al. This content is distributed under the terms of the Creative Commons Attribution 4.0 International license.

### Transposon library screen.

A total of 10^8^ CFUs of the E. faecalis OG1RF pooled transposon library was inoculated into 5 ml of THB and grown with aeration to an optical density of 600 nm (OD_600_) of 0.5. A total of 10^7^ CFUs of the library was spread onto a THB agar plate (10 replicates) containing 10 mM MgSO_4_ in the absence and presence of 10^6^ PFUs of VPE25 (MOI, 0.1). After overnight (O/N) incubation at 37°C, bacterial growth from the control and phage-containing plates was resuspended in 5 ml of phosphate-buffered saline (PBS). Genomic DNA was isolated from the input library and from three biological replicates of phage-exposed and unexposed samples using a ZymoBIOMICS DNA miniprep kit (Zymo Research), following the manufacturers protocol.

### Phage sensitivity and burst kinetic assays.

O/N cultures of E. faecalis were subcultured to a starting OD_600_ of 0.025 in 25 ml of THB. When the bacterial culture reached mid-logarithmic phase (OD_600_, ∼0.5), 10 mM MgSO_4_ and VPE25 (MOI of 0.1 or 10) were added. OD_600_ was monitored for ∼7 h. To investigate if phage progeny were produced and released from the bacterial cells upon VPE25 infection, 250 μl of culture was collected at different time points over the course of infection and thoroughly mixed with 1/3 volume of chloroform. The aqueous phase-containing phages was separated from the chloroform by centrifugation at 24,000 × *g* for 1 min, and the phage titer was determined using a THB agar overlay plaque assay. Data are presented as the average of three replicates with +/− standard deviation.

### Phage infection time course.

O/N cultures of E. faecalis were subcultured to a starting OD_600_ of 0.025 in 50 ml of THB. When the bacterial culture reached mid-logarithmic phase (OD_600_, ∼0.5), 10 mM MgSO_4_ and VPE25 (MOI of 10) were added. A total of 4 ml of cell suspension was pelleted from the uninfected and infected cultures after 0, 10, 20, and 40 minutes after VPE25 treatment. The pellets were washed with 4 ml of PBS three times, followed by a wash with 2 ml RNAlater (Invitrogen), and RNA isolation was performed as described above.

Further experimental details can be found in the supplemental Materials and Methods (Text S1).

10.1128/mBio.03120-19.1 TEXT S1 Supplementary Materials and Methods. Download Text S1, PDF file, 0.2 MB. Copyright © 2020 Chatterjee et al. 2020 Chatterjee et al. This content is distributed under the terms of the Creative Commons Attribution 4.0 International license.

### Data availability.

The RNA-Seq reads associated with this study have been deposited at the ArrayExpress database at EMBL-EBI under accession number E-MTAB-8546. Tn-Seq reads have been deposited at the European Nucleotide Archive under accession number PRJEB35492.
