# Supplementary figures and images for: Parallel Genomics Uncover Novel Enterococcal-Bacteriophage Interactions
Source: mBio. 2020 Mar 3;11(2):e03120-19. doi: 10.1128/mBio.03120-19 (PMC7064774; doi:10.1128/mBio.03120-19)

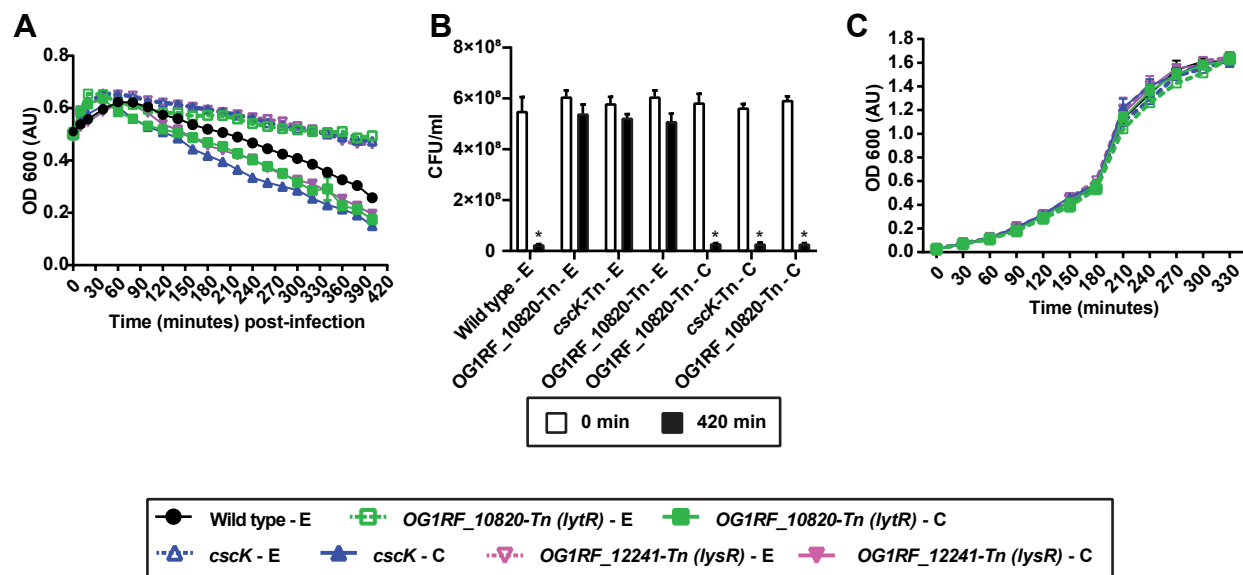

Supplement: FIG S1 [file mBio.03120-19-sf001.pdf]

**A**

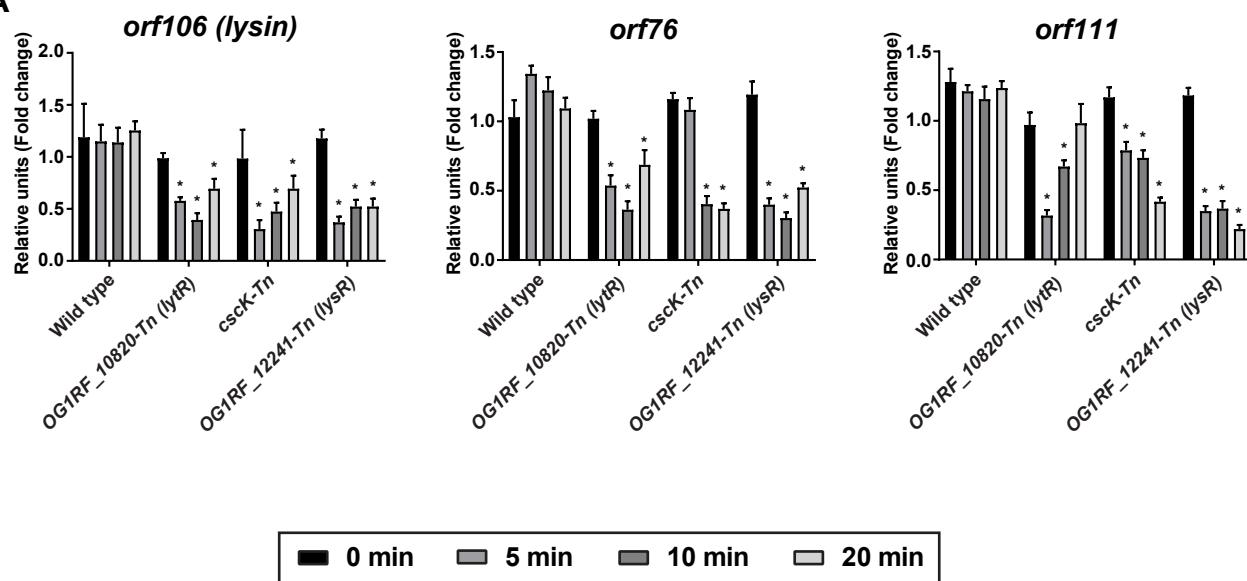

**B**

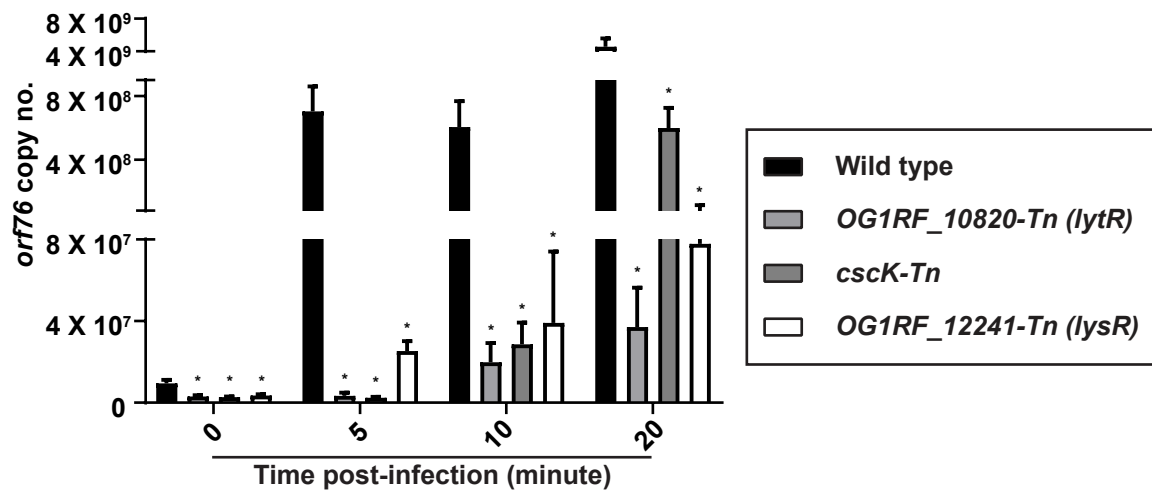

Supplement: FIG S2 [file mBio.03120-19-sf002.pdf]

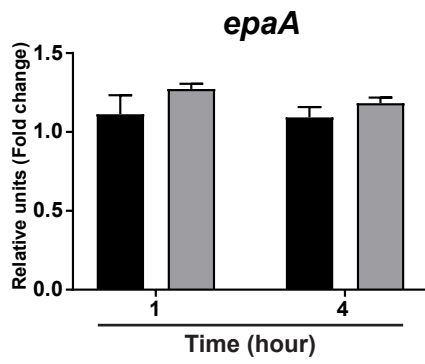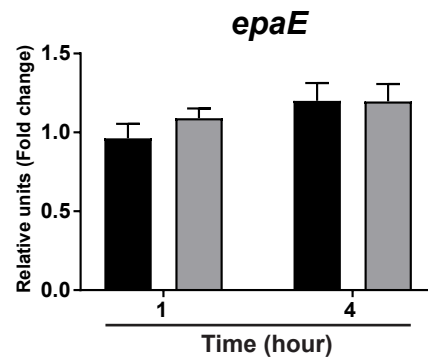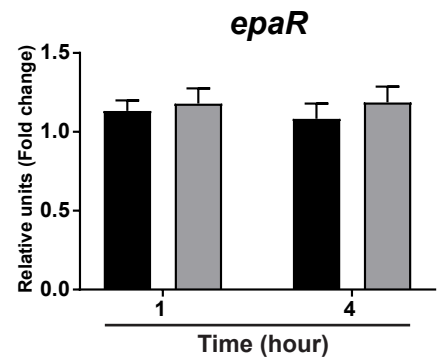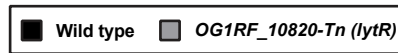

Supplement: FIG S3 [file mBio.03120-19-sf003.pdf]

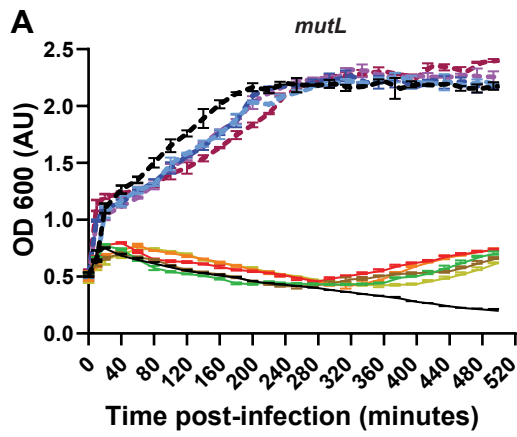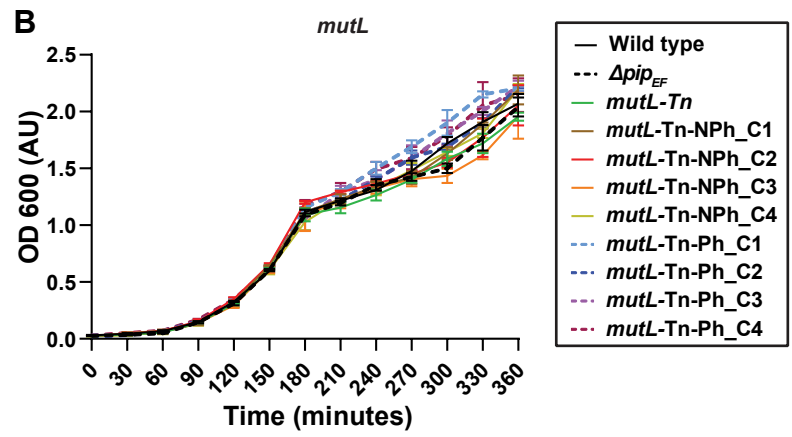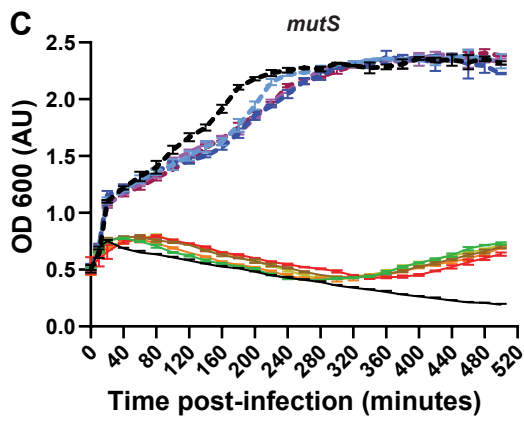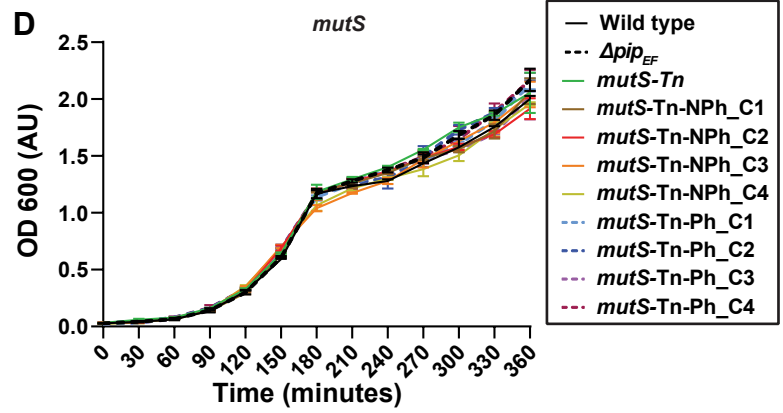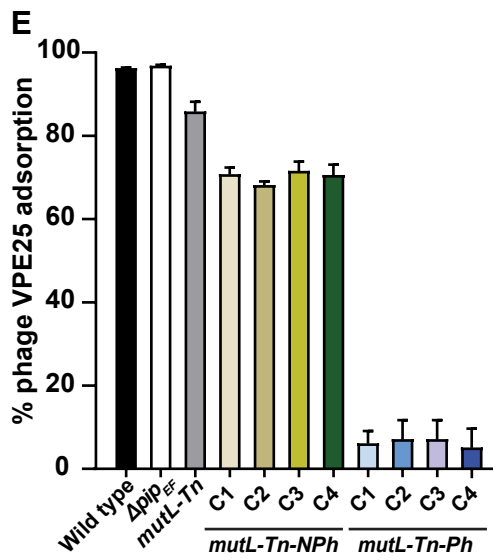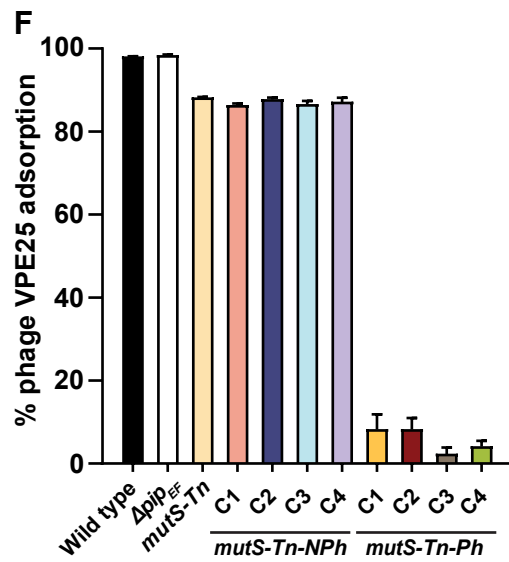

Supplement: FIG S4 [file mBio.03120-19-sf004.pdf]

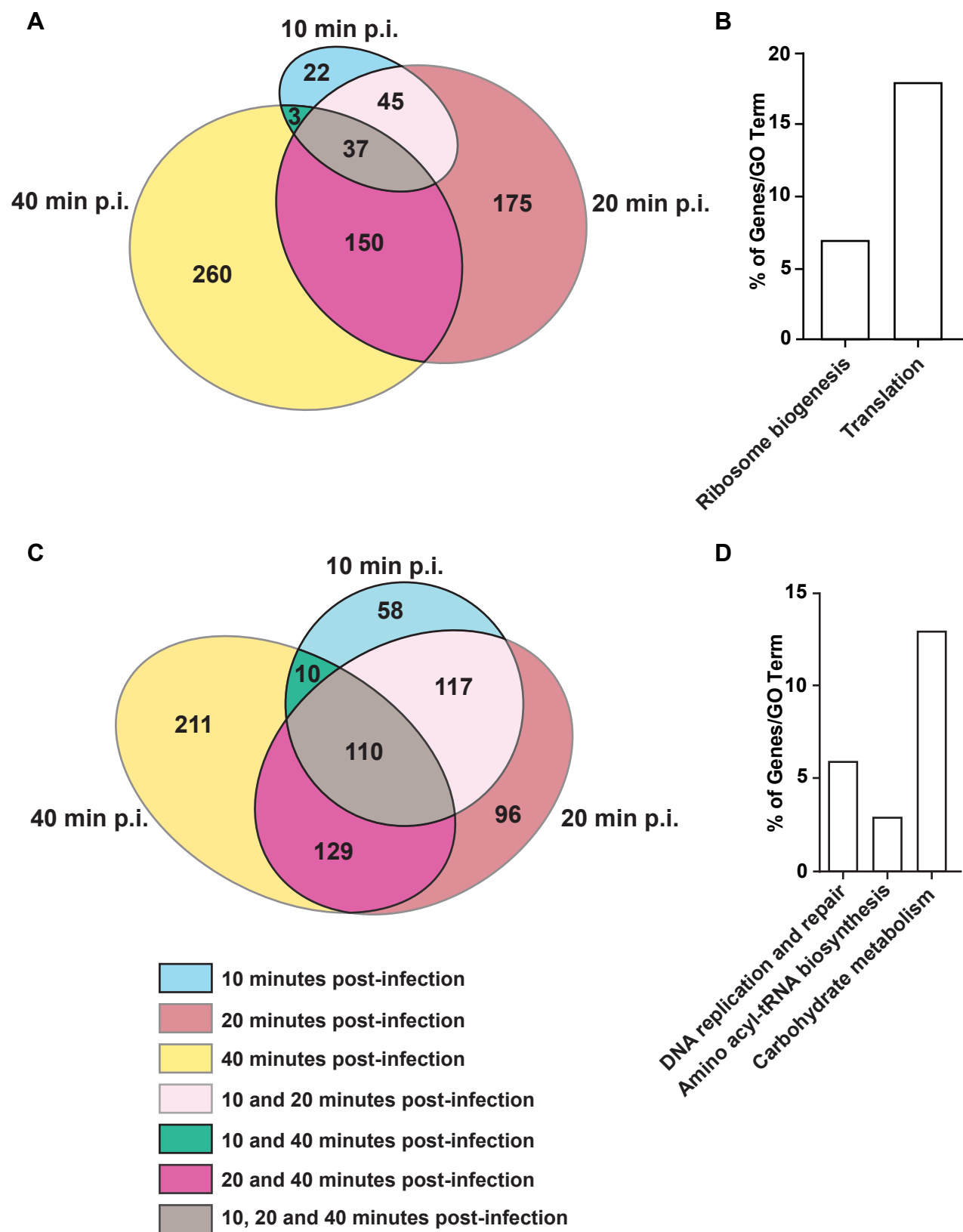

Supplement: FIG S5 [file mBio.03120-19-sf005.pdf]
