# Supplementary material for: Parallel Genomics Uncover Novel Enterococcal-Bacteriophage Interactions
Source: mBio. 2020 Mar 3;11(2):e03120-19. doi: 10.1128/mBio.03120-19 (PMC7064774; doi:10.1128/mBio.03120-19)
Supplement: FIG S6 [file mBio.03120-19-sf006.pdf]

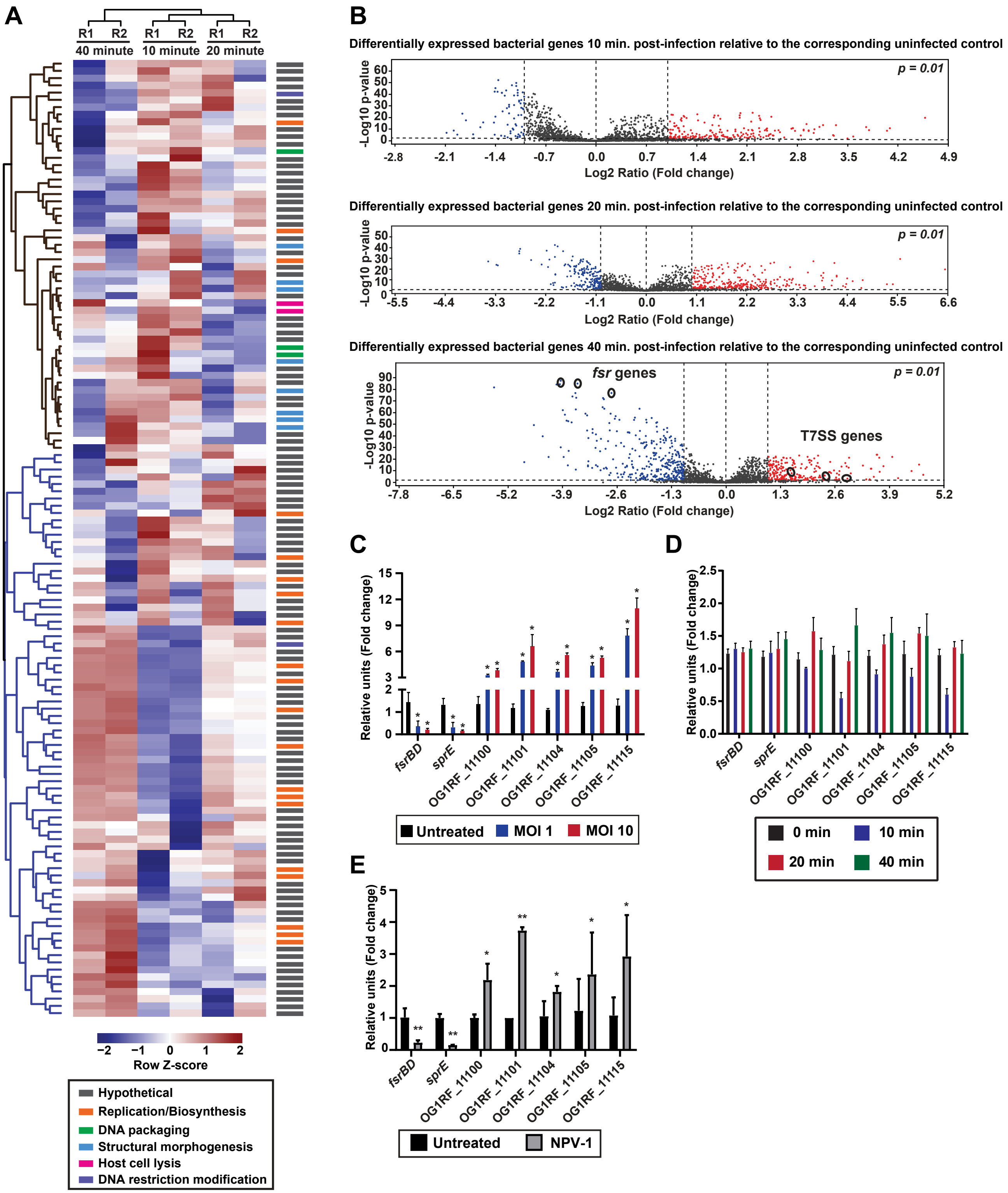
A
R1 R2 R1 R2 R1 R2
40 minute 10 minute 20 minute
−2 −1 0 1 2
Row Z-score
Hypothetical
Replication/Biosynthesis
DNA packaging
Structural morphogenesis
Host cell lysis
DNA restriction modification
B
Differentially expressed bacterial genes 10 min. post-infection relative to the corresponding uninfected control
p = 0.01
-Log10 p-value
Log2 Ratio (Fold change)
Differentially expressed bacterial genes 20 min. post-infection relative to the corresponding uninfected control
Differentially expressed bacterial genes 40 min. post-infection relative to the corresponding uninfected control
fsr genes
T7SS genes
C
Relative units (Fold change)
fsrBD
sprE
OG1RF_11100
OG1RF_11101
OG1RF_11104
OG1RF_11105
OG1RF_11115
Untreated
MOI 1
MOI 10
D
0 min
10 min
20 min
40 min
E
NPV-1
