# Supplementary material for: Parallel Genomics Uncover Novel Enterococcal-Bacteriophage Interactions
Source: mBio. 2020 Mar 3;11(2):e03120-19. doi: 10.1128/mBio.03120-19 (PMC7064774; doi:10.1128/mBio.03120-19)
Supplement: TABLE S3 [file mBio.03120-19-st003.pdf]

Table S3. Bacterial strains, phages, plasmids and primers used in this study.

Strains, phages, plasmids and primers	Characteristics and/or description	Reference /Source
Enterococcus faecalis		
OG1RF	Human oral isolate; Rf^R, Fa^R	(1)
Δpip_{EF}	OG1RF background with a deletion of OG1RF_10588 (PIP)	(2)
OG1RF_10820-Tn	*E. faecalis* OG1RF *OG1RF_10820* (LytR family response regulator) transposon mutant (Genomic position: 860054). Rf^R, Fa^R, Cm^R	(3)
cscK-Tn	*E. faecalis* OG1RF OG1RF_10951 (fructosekinase) transposon mutant (Genomic position: 993104). Rf^R, Fa^R, Cm^R	(3)
OG1RF_12241-Tn	*E. faecalis* OG1RF OG1RF_12241 (LysR family transcriptional regulator) transposon mutant (Genomic position: 2363777). Rf^R, Fa^R, Cm^R	(3)
OG1RF_11710-Tn	*E. faecalis* OG1RF OG1RF_11710 (O-antigen polymerase) transposon mutant (Genomic position: 1789261). Rf^R, Fa^R, Cm^R	(3)
OG1RF_11714-Tn	*E. faecalis* OG1RF OG1RF_11714 (group 2 glycosyl transferase) transposon mutant (Genomic position: 1793746). Rf^R, Fa^R, Cm^R	(3)
OG1RF_11715-Tn	*E. faecalis* OG1RF OG1RF_11715 (*epaOX*, glycosyltransferase) transposon mutant (Genomic position: 1794475). Rf^R, Fa^R, Cm^R	(3)
ΔepaOX	JD102; *E. faecalis* OG1RF *ΔepaOX* (Δ*OG1RF_11715,* glycosyltransferase) markerless deletion. Rf^R, Fa^R	(4)
OG1RF_12434-Tn	*E. faecalis* OG1RF OG1RF_12434 (*mutL*) locus transposon mutant (Genomic position: 2567339), Rf^R, Fa^R, Cm^R	(3)
OG1RF_12435-Tn	*E. faecalis* OG1RF OG1RF_12435 (*mutS*) locus transposon mutant (Genomic position: 2569574), Rf^R, Fa^R, Cm^R	(3)
Wild type - E	OG1RF strain carrying pAT28 empty vector	This study

Δ*pip*$_{EF}$ - E	Δ*pip*$_{EF}$ strain carrying pAT28 empty vector	This study
OG1RF_10820-Tn - E	OG1RF_10820 transposon mutant carrying pAT28 empty vector. Rf^R, Fa^R, Cm^R,Sp^R	This study
cscK-Tn - E	OG1RF_10951 transposon mutant carrying pAT28 empty vector. Rf^R, Fa^R, Cm^R, Sp^R	This study
OG1RF_12241-Tn - E	OG1RF_12241 transposon mutant carrying pAT28 empty vector. Rf^R, Fa^R, Cm^R, Sp^R	This study
OG1RF_10820-Tn - C	OG1RF_10820 transposon mutant carrying pAT28 complementation vector. Rf^R, Fa^R, Cm^R,Sp^R	This study
cscK-Tn - C	OG1RF_10951 transposon mutant carrying pAT28 complementation vector. Rf^R, Fa^R, Cm^R, Sp^R	This study
OG1RF_12241-Tn - C	OG1RF_12241 transposon mutant carrying pAT28 complementation vector. Rf^R, Fa^R, Cm^R, Sp^R	This study
mutL-Tn - E	OG1RF_12434 (*mutL*) locus transposon mutant carrying pAT28 empty vector. Rf^R, Fa^R, Cm^R, Sp^R	This study
mutS-Tn - E	OG1RF_12435 (*mutS*) locus transposon mutant carrying pAT28 empty vector. Rf^R, Fa^R, Cm^R, Sp^R	This study
mutL-Tn - C	OG1RF_12434 (*mutL*) locus transposon mutant carrying pAT28 complementation vector. Rf^R, Fa^R, Cm^R, Sp^R	This study
mutS-Tn - C	OG1RF_12435 (*mutS*) locus transposon mutant carrying pAT28 complementation vector. Rf^R, Fa^R, Cm^R, Sp^R	This study
mutL-Tn-NPh_C1	*mutL*-Tn colony # 1 selected from O/N THB agar plate. Rf^R, Fa^R, Cm^R	This study
mutL-Tn-NPh_C2	*mutL*-Tn colony # 2 selected from O/N THB agar plate. Rf^R, Fa^R, Cm^R	This study
mutL-Tn-NPh_C3	*mutL*-Tn colony # 3 selected from O/N THB agar plate. Rf^R, Fa^R, Cm^R	This study
mutL-Tn-NPh_C4	*mutL*-Tn colony # 4 selected from O/N THB agar plate. Rf^R, Fa^R, Cm^R	This study
mutL-Tn-Ph_C1	*mutL*-Tn colony # 1 selected from O/N THB agar plate containing VPE25. Rf^R, Fa^R, Cm^R	This study
mutL-Tn-Ph_C2	*mutL*-Tn colony # 2 selected from O/N THB agar plate containing VPE25. Rf^R, Fa^R, Cm^R	This study

mutL-Tn-Ph_C3	*mutL*-Tn colony # 3 selected from O/N THB agar plate containing VPE25. Rf^R, Fa^R, Cm^R	This study
mutL-Tn-Ph_C4	*mutL*-Tn colony # 4 selected from O/N THB agar plate containing VPE25. Rf^R, Fa^R, Cm^R	This study
mutS-Tn-NPh_C1	*mutS*-Tn colony # 1 selected from O/N THB agar plate. Rf^R, Fa^R, Cm^R	This study
mutS-Tn-NPh_C2	*mutS*-Tn colony # 2 selected from O/N THB agar plate. Rf^R, Fa^R, Cm^R	This study
mutS-Tn-NPh_C3	*mutS*-Tn colony # 3 selected from O/N THB agar plate. Rf^R, Fa^R, Cm^R	This study
mutS-Tn-NPh_C4	*mutS*-Tn colony # 4 selected from O/N THB agar plate. Rf^R, Fa^R, Cm^R	This study
mutS-Tn-Ph_C1	*mutS*-Tn colony # 1 selected from O/N THB agar plate containing VPE25. Rf^R, Fa^R, Cm^R	This study
mutS-Tn-Ph_C2	*mutS*-Tn colony # 2 selected from O/N THB agar plate containing VPE25. Rf^R, Fa^R, Cm^R	This study
mutS-Tn-Ph_C3	*mutS*-Tn colony # 3 selected from O/N THB agar plate containing VPE25. Rf^R, Fa^R, Cm^R	This study
mutS-Tn-Ph_C4	*mutS*-Tn colony # 4 selected from O/N THB agar plate containing VPE25. Rf^R, Fa^R, Cm^R	This study
Escherichia coli		
TG1	*[F' traD36 proAB lacIqZ ΔM15] supE thi-1 Δ(lac-proAB) Δ(mcrBhsdSM)5(rK - mK -)*	Lucigen
**Phage**		
VPE25	Siphoviridae; Wastewater isolate	(2)
NPV1	Siphoviridae; Wastewater isolate	(5)
**Plasmids**		
pAT28	*Escherichia coli* and in Gram-positive bacteria shuttle vector; Sp^R	(6)
pAT28: OG1RF_10820	pAT28 expressing OG1RF_10820 from the native promoter. Cloned into SacI/BamHI site. Sp^R	This study
pAT28: *cscK*	pAT28 expressing *cscK* from the native promoter. Cloned into SacI/BamHI site. Sp^R	This study

pAT28: OG1RF_12241	pAT28 expressing OG1RF_12241 from the native promoter. Cloned into SacI/BamHI site. Sp^R	This study
pAT28: *muL*	pAT28 expressing *mutL* from the native promoter. Cloned into SacI/XbaI site. Sp^R	This study
pAT28: *mutS*	pAT28 expressing *mutS* from the native promoter. Cloned into SacI/EcoRI site. Sp^R	This study
**Primers**		
lytR-F	NNNNNNGAGCTCACAGTTATTTTTTTTGTTTGGTGTTAC; Forward primer to generate pAT28:*OG1RF_10820*; SacI site	This study
lytR-R	NNNNNNGGATCCGAATCTATGTTATAACTAACATCATTG; Reverse primer to generate pAT28:*OG1RF_10820*; BamHI site	This study
cscK-F	NNNNNNGAGCTCTCATTGTTAGATAACCGTGAATGCTC; Forward primer to generate pAT28:*cscK*; SacI site	This study
cscK-R	NNNNNNGGATCCTCATTCTTAGCCATTTCCTTCAAAT; Reverse primer to generate pAT28:*cscK*; BamHI site	This study
lysR-F	NNNNNNGAGCTCTCGCCTCCTTTTGCTAAATTTATCG; Forward primer to generate pAT28:*OG1RF_12241*; SacI site	This study
lysR-R	NNNNNNGGATCCAGTATTCAACGACCTATTATTCAG; Reverse primer to generate pAT28:*OG1RF_12241*; BamHI site	This study
mutS-F	NNNNNNGAGCTCCCTCCTAACCGGTTCGGTTCCTAATTAGA; Forward primer to generate pAT28:*mutS*; SacI site	This study
mutS-R	NNNNNNGAATTCGAAGAAAAGTTAATCTCGGATCATTAAGAGG; Reverse primer to generate pAT28:*mutS*; EcoRI site	This study
mutL-F	NNNNNNGAGCTCAATGATGACGCCTCGTTTCTTTTTAATGGG; Forward primer to generate pAT28:*mutL*; SacI site	This study
mutL-R	NNNNNNTCTAGAAGGAACCGAACCGGTTAGGAGGTCGTGAAATGG; Reverse primer to generate pAT28:*mutL*; XbaI site	This study

RT-*fsrA* -F	GCAGGATTTGAGGTTGCTAAAG; qPCR forward primer for *fsrA*	This study
RT-*fsrA* -R	GAGCTGAAACCATATATTTGTAGGAAG; qPCR reverse primer for *fsrA*	This study
RT-*fsrBD* -F	TCTTCTGTGAGCTTACCGTTTATT; qPCR forward primer for *fsrBD*	This study
RT-*fsrBD* -R	CAGGTTCAATTGCTGTTCCTTG; qPCR reverse primer for *fsrBD*	This study
RT-*fsrC* -F	TAACGAAGAGCTAGCGATGTTT; qPCR forward primer for *fsrC*	This study
RT-*fsrC* -R	GGTTGGTGCAATCGTTTCTTC; qPCR reverse primer for *fsrC*	This study
RT-*gelE* -F	CGCCATCACTAGCGACATTA; qPCR forward primer for *gelE*	This study
RT-*gelE* -R	GGCATCCCTCGATCATCAATAC; qPCR reverse primer for *gelE*	This study
RT-*sprE* -F	AGAAGACAAGAAGTGGCAGATAC; qPCR forward primer for spr*E*	This study
RT-*sprE* -R	CAACAACAAAGCCTGTTCCTAAA; qPCR reverse primer for spr*E*	This study
RT-10296 -F	GCTTCACCAGGCTCCATTATAG; qPCR forward primer for OG1RF_10296	This study
RT-10296 -F	TGACTGCGGATACTGCAAAG; qPCR reverse primer for OG1RF_10296	This study
RT-10297 -F	TGTGTTGAGCCCGCTTATATC; qPCR forward primer for OG1RF_10297	This study
RT-10297 -F	TGGCTTCTTCTGTTCCATGAG; qPCR reverse primer for OG1RF_10297	This study
RT-10875 -F	GACTGTATTACGCTTCGCTAAAG; qPCR forward primer for OG1RF_10875	This study
RT-10875 -R	CCACATTGAACTGCCCATAAA; qPCR reverse primer for OG1RF_10875	This study
RT-10876 -F	TGTGGGCTAAGCTAATGAATTT; qPCR forward primer for OG1RF_10876	This study

RT-10876 -R	AGTTCTCCTGAATCTATCCAAGTA; qPCR reverse primer for OG1RF_10876	This study
RT-*eutB* -F	CAATGGAAGCGCGTTGTTATG; qPCR forward primer for *eutB*	This study
RT-*eutB* -R	AGCGCGGATGACTTGTTT; qPCR reverse primer for *eutB*	This study
RT-*eutC* -F	CAGCTGCTATCGAAGCGAATA; qPCR forward primer for *eutC*	This study
RT-*eutC* -R	TTGCTGGCACACGACAATA; qPCR reverse primer for *eutC*	This study
RT-*eutH*-F	TAGCTGGCGCTTTCTGTTTAG; qPCR forward primer for *eutH*	This study
RT-*eutH* -R	AGCCGCTACTTCATTCATTCC; qPCR reverse primer for *eutH*	This study
RT-*epaA* -F	GCAGGAGGACTTGCAATCTA; qPCR forward primer for *epaA*	This study
RT-*epaA* -R	ACCATTCCACCAGCCAAA; qPCR reverse primer for *epaA*	This study
RT-*epaE* -F	AGGGATCAAACCGTCAGAAC; qPCR forward primer for *epaA*	This study
RT-*epaE* -R	GGCCCATTACCTCAACAGAA; qPCR reverse primer for *epaA*	This study
RT- *epaOX* -F	CCACGTTAACTCGTGCAGTA; qPCR forward primer for *epaOX*	This study
RT- *epaOX* -R	CCACTGTCATCAGAAGAACCA; qPCR reverse primer for *epaOX*	This study
RT- 11714 -F	GGAAGCATTACCGCTTCTAAGT; qPCR forward primer for OG1RF_11714	This study
RT- 11714 -R	ATGCAAGGTCGCTATATTCTGG; qPCR reverse primer for OG1RF_11714	This study
RT- 11710 -F	GTGTATGGTTTGTCTTAGCGTTAG; qPCR forward primer for OG1RF_11710	This study
RT- 11710 -R	TCGGCCTTCATAGAAACCTATAC; qPCR reverse primer for OG1RF_11710	This study

RT- 11100 -*F*	AAGGGCAAGCATTTCAAGCG; qPCR forward primer for OG1RF_11100	This study
RT- 11100 -*R*	TCTTGACGGTCACGTTCTGC; qPCR reverse primer for OG1RF_11100	This study
RT- 11101 -*F*	CCAATGGCTTGGCAACTGAC; qPCR forward primer for OG1RF_11101	This study
RT- 11101 -*R*	GCGAACGAACGTGCATTTTG; qPCR reverse primer for OG1RF_11101	This study
RT- 11104 -*F*	GGGAATGGCACCCTGAAAGA; qPCR forward primer for OG1RF_11104	This study
RT- 11104 -*R*	CTTCGCGCTTGGCTTTTTGA; qPCR reverse primer for OG1RF_11104	This study
RT- 11105 -*F*	TTGGAAAGGTGGCGGAATAG; qPCR forward primer for OG1RF_11105	This study
RT- 11105 -*R*	TCTGCTTTGATACTGGCTAAGG; qPCR reverse primer for OG1RF_11105	This study
RT- 11109 -*F*	GCTTTGGAGAACGCTGAACG; qPCR forward primer for OG1RF_11109	This study
RT- 11109 -*R*	TTTTGACAGTCTTGCGCTCG; qPCR reverse primer for OG1RF_11109	This study
RT- 11115 -*F*	CTCAACCGGATCGTGCTTATT; qPCR forward primer for OG1RF_11115	This study
RT- 11115 -*R*	CCTTGGTAGCGAATGGATCATAG; qPCR reverse primer for OG1RF_11115	This study
RT-16S -F	CGCTTCTTTCCTCCCGAGT; qPCR forward primer 16S rRNA gene	This study
RT-16S -F	GCCATGCGGCATAAACTG; qPCR reverse primer 16S rRNA gene	This study

Cm^R - chloramphenicol resistant; Rf^R - rifampicin resistance; Fa^R - fusidic acid resistance; SpR - spectinomycin resistance; Restriction enzyme sites are underlined

References

1. Bourgogne A, Garsin DA, Qin X, Singh KV, Sillanpaa J, Yerrapragada S, Ding Y, Dugan-Rocha S, Buhay C, Shen H, Chen G, Williams G, Muzny D, Maadani A, Fox KA, Gioia J, Chen L, Shang Y, Arias CA, Nallapareddy SR, Zhao M, Prakash VP, Chowdhury S, Jiang H, Gibbs RA, Murray BE, Highlander SK, Weinstock GM. 2008. Large scale variation in *Enterococcus faecalis* illustrated by the genome analysis of strain OG1RF. Genome Biol 9:R110. doi:10.1186/gb-2008-9-7-r110.

2. Duerkop BA, Huo W, Bhardwaj P, Palmer KL, Hooper LV. 2016. Molecular basis for lytic bacteriophage resistance in enterococci. MBio 7. doi:10.1128/mBio.01304-16.

3. Dale JL, Beckman KB, Willett JLE, Nilson JL, Palani NP, Baller JA, Hauge A, Gohl DM, Erickson R, Manias DA, Sadowsky MJ, Dunny GM. 2018. Comprehensive functional analysis of the *Enterococcus faecalis* core genome using an ordered, sequence-defined collection of insertional mutations in strain OG1RF. mSystems 3. doi:10.1128/mSystems.00062-18.

4. Dale JL, Cagnazzo J, Phan CQ, Barnes AM, Dunny GM. 2015. Multiple roles for *Enterococcus faecalis* glycosyltransferases in biofilm-associated antibiotic resistance, cell envelope integrity, and conjugative transfer. Antimicrob Agents Chemother 59:4094-105. doi:10.1128/AAC.00344-15.

5. Trotter KM, Dunny GM. 1990. Mutants of *Enterococcus faecalis* deficient as recipients in mating with donors carrying pheromone-inducible plasmids. Plasmid 24:57-67.

6. Trieu-Cuot P, Carlier C, Poyart-Salmeron C, Courvalin P. 1990. A pair of mobilizable shuttle vectors conferring resistance to spectinomycin for molecular cloning in

Escherichia coli and in Gram-positive bacteria. Nucleic Acids Res 18:4296.
doi:10.1093/nar/18.14.4296.
