# Supplementary material for: Parallel Genomics Uncover Novel Enterococcal-Bacteriophage Interactions
Source: mBio. 2020 Mar 3;11(2):e03120-19. doi: 10.1128/mBio.03120-19 (PMC7064774; doi:10.1128/mBio.03120-19)
Supplement: TEXT S1 [file mBio.03120-19-s0001.pdf]

Supplementary Materials and Methods

Transposon library sequencing. Library preparation and sequencing was performed by the Microarray and Genomics Core at the University of Colorado Anschutz Medical Campus. A detailed protocol is described by Dale *et al.* (1). Briefly, 100 ng of genomic DNA was sheared to approximately 400 bp fragments and processed through the Illumina TruSeq Nano library enrichment kit. 9 ng of each normalized library was used as PCR template to enrich for the *mariner* transposon junctions using a transposon-specific primer (*mariner*-seq) and the Illumina P7 primer (16 cycles of amplification). The enrichment PCR products were diluted 1:100, and 10 µl was used as template for an indexing PCR of 9 cycles of amplification (TruSeq P5 indexing primer + P7 primer). The final libraries had unique combinations of P5 and P7 indexes suitable for multiplexed sequencing. Sequencing was performed using Illumina NovaSeq 6000 in 150 base paired-end format. Illumina adapter trimming, read mapping to the *E. faecalis* OG1RF reference sequence (NC_017316.1) and statistical analysis of differentially abundant transposon mutants were performed using previously published scripts found here https://github.com/dunnylabumn/Ef_OG1RF_tnseq (1).

RNA sequencing and bioinformatics analysis. RNASeq libraries were constructed using the Ribo Depleted library construction kit for Gram-positive Bacteria (Illumina). Sequencing was performed using Illumina NovaSeq 6000 in 150 base paired-end format. RNASeq data were analyzed using Geneious R11. Sequencing reads were mapped to the *E. faecalis* OG1RF (NC_017316.1) and VPE25 (LT546030.1) genomes using Bowtie2. Gene expression values were calculated by reads per kilobase per million which normalizes the raw count by transcript length and sequence depth. Differential expression between two samples was determined using the default Geneious R11 method with median ratios across all the transcripts as the normalization scale. Genes with a fold change of ≥ 2.0 and P values ≤ 0.05 were considered significantly differentially expressed. Blast2GO basic tool was used to assign gene ontology (GO) terms to *E. faecalis* OG1RF genes (2). KEGG pathway analysis was performed using

the KEGG annotation server (3). The ClueGo plug-in for Cytoscape 3.6.1 (4, 5) was used to visualize gene clustering based on GO terms and KEGG annotations.

Bacterial growth curves. 25 ml of THB was inoculated with O/N cultures of *E. faecalis* to obtain a starting OD_{600} of 0.025. Cultures were incubated at 37° C with aeration. OD_{600} was measured periodically for ~7 hours. Growth curves are presented as the average of three biological replicates.

Bacterial spot assay on phage agar plates. O/N bacterial cultures were pelleted and resuspended in SM-plus buffer (100 mM NaCl, 50 mM Tris-HCl, 8 mM MgSO4, 5 mM CaCl2 [pH 7.4]) and normalized to an OD600 of 1.0. 10-fold serial dilutions of the bacterial cultures were spotted onto THB agar plates with or without 5x106 PFU/ml of VPE25. The plates were incubated at 37°C O/N.

Efficiency of plating (EOP) assay. Phage titer was determined using a THB agar overlay plaque assay against the wild type strain. Serial dilutions of phage suspensions starting from 10^6 PFU/ml were mixed with O/N cultures of wild type or mutant strains to perform THB agar overlay plaque assays. PFU/ml were determined after O/N incubation at 37° C and compared with the starting PFU/ml to obtain percent efficiency of plating. Data are presented as the average of three replicates with +/- standard deviation.

Phage adsorption assay. A bacterial culture grown O/N was pelleted at 3,220 × *g* for 10 min and resuspended to 10^8 CFU/ml in SM-plus buffer. The cell suspensions were mixed with phages at an MOI of 0.1 and incubated at room temperature without agitation for 10 min. The bacterium-phage suspensions were centrifuged at 24,000 × *g* for 1 min, and the supernatant was collected and phages were enumerated by a plaque assay. SM-plus buffer with phage only (no bacteria) served as a control. Percent adsorption was determined as follows: $[(PFU_{control} - PFU_{test\ supernatant})/PFU_{control}] \times 100$. Data are presented as the average of three replicates +/- standard deviation.

Complementation of Tn mutants. All PCR reactions used for cloning were performed with high fidelity KOD Hot Start DNA Polymerase (EMD Millipore). Approximately 100 bp of upstream flanking DNA and the coding regions of OG1RF_10820 (*lytR*), OG1RF_10951 (*cscK*), OG1RF_12241 (*lysR*), OG1RF_12435 (*mutS*) and OG1RF_12434 (*mutL*) were cloned into the shuttle vector pAT28 (6). The primer sequences and restriction enzymes used for cloning are listed in Table S4. Plasmids were introduced into electrocompetent *E. faecalis* cells as previously described (7).

RNA extraction and quantitative PCR. RNA was extracted from uninfected or VPE25 infected *E. faecalis* by using an RNeasy Mini Kit (Qiagen) with the following modifications. Cell pellets were incubated in 100 µL of 15mg/ml lysozyme (Amersco) for 30 min at room temperature. 700 µl of RLT buffer containing β-mercaptoethanol (manufacturers recommended concentration) was added and the samples were bead beat in Lysing Matix B tubes (MP Bio) at 45 sec intervals for a total time of 4.5 min. Debris was centrifuged at 24,000 × *g* for 1 min and the supernatant was transferred to a fresh tube. 590 µL of 80% ethanol per 760 µL supernatant was added and the entire volume was loaded onto an RNeasy column following the standard Qiagen RNA purification protocol. cDNA was synthesized from 1 µg of total RNA using qScript cDNA SuperMix (QuantaBio) (25°C for 5 minutes, 42°C for 30 minutes and 85°C for 5 minutes). Transcript levels were analyzed by qPCR using PowerUp™ SYBR Green Master Mix (Applied Biosystems) and transcript abundances were normalized to the 16S rRNA transcripts. VPE25 orf_76 copy number was determined by qPCR using orf_76 cloned into pCR4-TOPO™ TA cloning vector (Invitrogen) as a standard. All data are represented as the average of three replicates +/- the standard deviation.

Statistical analysis. Statistical tests were performed using GraphPad – Prism version 8.2.1. For bacterial growth assays in the presence and absence of phage, mutant *E. faecalis* was compared to the wild type using two-way analysis of variance (ANOVA). For qPCR and phage adsorption assays, unpaired Student's t-tests were used. GO term linkages were assigned through ClueGO using a kappa

statistical test ($p < 0.05$), which measures the consensus between qualitative categorical items. *P* values are indicated in the figure legends.

References

1. Dale JL, Beckman KB, Willett JLE, Nilson JL, Palani NP, Baller JA, Hauge A, Gohl DM, Erickson R, Manias DA, Sadowsky MJ, Dunny GM. 2018. Comprehensive functional analysis of the *Enterococcus faecalis* core genome using an ordered, sequence-defined collection of insertional mutations in strain OG1RF. mSystems 3. doi:10.1128/mSystems.00062-18.

2. Gotz S, Garcia-Gomez JM, Terol J, Williams TD, Nagaraj SH, Nueda MJ, Robles M, Talon M, Dopazo J, Conesa A. 2008. High-throughput functional annotation and data mining with the Blast2GO suite. Nucleic Acids Res 36:3420-35. doi:10.1093/nar/gkn176.

3. Moriya Y, Itoh M, Okuda S, Yoshizawa AC, Kanehisa M. 2007. KAAS: an automatic genome annotation and pathway reconstruction server. Nucleic Acids Res 35:W182-5. doi:10.1093/nar/gkm321.

4. Shannon P, Markiel A, Ozier O, Baliga NS, Wang JT, Ramage D, Amin N, Schwikowski B, Ideker T. 2003. Cytoscape: a software environment for integrated models of biomolecular interaction networks. Genome Res 13:2498-504. doi:10.1101/gr.1239303.

5. Bindea G, Mlecnik B, Hackl H, Charoentong P, Tosolini M, Kirilovsky A, Fridman WH, Pages F, Trajanoski Z, Galon J. 2009. ClueGO: a Cytoscape plug-in to decipher functionally grouped gene ontology and pathway annotation networks. Bioinformatics 25:1091-3. doi:10.1093/bioinformatics/btp101.

6. Trieu-Cuot P, Carlier C, Poyart-Salmeron C, Courvalin P. 1990. A pair of mobilizable shuttle vectors conferring resistance to spectinomycin for molecular cloning in *Escherichia coli* and in Gram-positive bacteria. Nucleic Acids Res 18:4296. doi:10.1093/nar/18.14.4296.

7. Chatterjee A, Johnson CN, Luong P, Hullahalli K, McBride SW, Schubert AM, Palmer KL, Carlson PE, Jr., Duerkop BA. 2019. Bacteriophage resistance alters antibiotic-mediated intestinal expansion of enterococci. Infect Immun 87. doi:10.1128/IAI.00085-19.
